# Developmental air pollution exposure augments airway hyperreactivity, alters transcriptome, and DNA methylation in female adult progeny

**DOI:** 10.1038/s42003-025-07835-0

**Published:** 2025-03-08

**Authors:** Razia Zakarya, Yik Lung Chan, Baoming Wang, Andrew Thorpe, Single-Cell Mouse Lung Disease Atlas Consortium, Sobia Idrees, Fia S. Boedijono, Alen Faiz, Philip M. Hansbro, Dikaia Xenaki, Kin Fai Ho, Hai Guo, Hui Chen, Brian G. Oliver, Christopher O’Neill

**Affiliations:** 1 School of Life Sciences, University of Technology Sydney, Sydney, Australia; 2 Epigenetics of Chronic Disease Group, Woolcock Institute of Medical Research, Macquarie University, Sydney, Australia; 3 Respiratory Cell and Molecular Biology Group, Woolcock Institute of Medical Research, Macquarie University, Sydney, Australia; 4 Jockey Club School of Public Health and Primary, The Chinese University of Hong Kong, Hong Kong Special Administrative Region of the People’s Republic of China, Hong Kong, China; 5 Air Quality Studies, Department of Civil and Environmental Engineering, The Hong Kong Polytechnic University, Hong Kong, China; 6 Centre for Inflammation, Centenary Institute and University of Technology Sydney, Faculty of Science, School of Life Sciences, Sydney, Australia; 7 University of Technology Sydney, Respiratory Bioinformatics and Molecular Biology (RBMB), School of Life Sciences, Sydney, Australia; 8 University of Groningen, University Medical Center Groningen, Groningen Research Institute for Asthma and COPD (GRIAC), Groningen, The Netherlands

**Keywords:** DNA methylation, Transcriptomics, Asthma

## Abstract

Maternal exposure to particulate air pollution increases the incidence and severity of asthma in offspring, yet the mechanisms for this are unclear. Known susceptibility loci are a minor component of this effect. We interrogate a mouse allergic airway disease model to assess epigenetic associations between maternal air pollution exposure and asthma responses in offspring. Maternal air pollution exposure increased allergic airway disease severity in adult offspring associated with a suppressed transcriptomic response. Control progeny showed differential expression of 2842 genes across several important pathways, whilst air pollutant progeny showed an 80% reduction in differentially expressed genes and abrogation of many pathway associations. Whole genome CpG methylome analysis following allergen challenge detected differential methylation regions across the genome. Differentially methylated regions were markedly reduced in air pollutant offspring, and this was most evident in intronic regions and some transposable element classes. This study shows that asthma in adult offspring of PM_2.5_ exposed mothers had a markedly repressed transcriptomic response, a proportion of which was associated with identifiable changes in the lung’s methylome. The results point to an epigenetic contribution to the severity of asthma in offspring of mothers exposed to particulate air pollution.

## Abstract (web summary)

A preclinical murine asthma model shows that exposure to air pollution particulates during development causes worsened airway hyperreactivity and points to a transcriptomic and DNA methylation memory of developmental environment insult in adult offspring.

## Introduction

Asthma is a chronic respiratory disease that caused 455,000 deaths and was experienced by 262 million people worldwide in 2019^1^. Asthma results in airway obstruction and wheezing, associated with airway hyperresponsiveness (AHR) and commonly a Type-2 eosinophilic inflammation response^2^, although other inflammatory endotypes also manifest across patients^2^. AHR is defined as a heightened contractile response of the airway to non-specific stimuli, including chemicals such as histamine and methacholine^3^, thereby inducing excessive bronchoconstriction and subsequent increased airway resistance. Asthma has a heritable component, with estimates ranging from 40 to 90%^4–8^. While some susceptibility loci have been identified^9^, such loci are also present in healthy populations and only explain a small proportion of prevalence^10–12^. This relatively low concordance between definable genetic traits and the susceptibility to asthma suggests that other shared factors may contribute to its heritability.

A number of environmental factors are shown to be associated with asthma incidence and severity, including aeroallergens (e.g., house dust mites, pet dander, cockroach, mould and pollen^13–16^) and components of air pollution, such as carbon monoxide (CO), sulfur dioxide (SO_2_), and particulate matter <2.5 μm in diameter (PM_2.5_)^17,18^. PM_2.5_ within air is an important factor linked to increased asthma incidence^19,20^, severity^21^, and hospitalisations^22^ in humans and as such, is the focus of this study. Associations between maternal PM_2.5_ exposure and increased asthma incidence in offspring have also been reported. Mother–child dyads with spatiotemporal PM_2.5_ exposure estimation during embryonic development showed an association between maternal exposure to PM_2.5_ and increased risk of developing asthma in children^23,24^. These studies point to the risks of maternal exposure to air pollution during pregnancy on the lung function of her progeny and highlight PM_2.5_ as an important component of this effect.

Mouse models of allergic airway disease (AAD) have been developed to interrogate the mechanisms by which environmental insults, including PM_2.5_, affect asthma outcomes. The basis of these models involves the sensitisation and challenge of animals to allergens (e.g., ovalbumin^25,26^ (OVA) and house dust mite^26^) followed by the assessment of airway responsiveness by exposure to spasmogens such as methacholine^25,27^. In the context of direct exposure to PM_2.5_, it has been shown^27^ that a single insult of PM_2.5_ induced higher airway hyperresponsiveness (AHR), with and without OVA challenge. It should be noted that this occurred in a PM_2.5_ dose-dependent manner, with significant differences at the highest concentration compared to low concentration and non-PM_2.5_ exposed animals, and that OVA-challenged animals demonstrated a trend towards higher AHR, signifying an interaction effect between OVA challenge and PM_2.5_ on AHR. It was further shown^28^ that PM_2.5_ exposure and OVA challenge induced increased pulmonary inflammation and decreased the expression of the epigenetic modifiers DNA methyltransferase-1 (*Dnmt1*) and 3b (*Dnmt3b*).

Maternal PM_2.5_ exposure during development is reported to affect pulmonary outcomes in her progeny across a range of models. A single maternal bronchial exposure to PM_2.5_ induced aberrant tracheal development in the offspring^29^. Maternal PM_2.5_ exposure over numerous time points over development induced lung function changes in tissue elastance, decreased alveolar density, and broad changes in gene expression^30,31^. Gene set enrichment analysis (GSEA) showed key pathways related to respiratory distress, lung disease, and inflammation and immune response were affected^30^. Although maternal PM_2.5_ insult alone has been shown to have no effect on AHR^31^ in young offspring, other maternal models of PM_2.5_ exposure combined with an OVA challenge in adult offspring have reported lung function changes associated with maternal PM_2.5_ exposure^32^.

These experimental models point to the embryo acquiring a form of molecular or functional memory of its mother’s environment during development that is expressed as abnormal postnatal lung function. Such ‘developmental memory’ has been reported as an important contributing factor to a wide range of chronic, non-communicable disease states and is recognised as the developmental origins of disease^33,34^. An important form of molecular memory of developmental events may be alterations in the state of an individual’s epigenome^35–37^.

The epigenome refers to mitotically heritable changes to chromatin structure and can alter patterns or levels of gene expression without altering DNA sequence. An individual’s epigenome is encoded at a number of important developmental transitions associated with cell lineage specification, and within a lineage the epigenome is considered to be mitotically heritable. However, unlike genetic polymorphisms, epigenetic modifications can permutate in response to an individual’s environment and exert robust phenotypic effects^38–40^. There are many epigenetic effectors, including DNA methylation (DNAm), which—in conjunction with its binding partners—has broad epigenetic effects on chromatin conformation, genomic integrity, and gene transcription^41–44^ through interactions with promoter regions, chromatin-modifying complexes, and other cis-regulatory mechanisms^45^.

DNAm occurs through the addition of a methyl moiety to the 5th carbon within cytosine, converting it to 5-methylcytosine (5mC), predominantly at CpG dinucleotides^46^. This reaction is mediated by DNA methyltransferases (DNMT1, DNMT3A, and DNMT3B) and can impact gene expression and disease outcome^45^. Investigations assessing clinical samples with methylation arrays have shown DNAm patterns that are unique to asthma^6,7,47,48^ and differential methylation at biologically relevant asthma gene targets^49^. Numerous DNAm epigenome-wide array studies (EWAS) on clinical samples with geocoded ambient air pollution exposure showed differential gene expression and CpG methylation associated with the extent of ambient air pollution in saliva^50^, peripheral whole blood^51,52^, cord blood^53,54^, and nasal epithelium^55^ from offspring. While array-based strategies provide power by facilitating economical screening of large numbers of samples, they are limited in they do not detect differentially methylated regions (DMRs) outside those predefined specifically to each array design. Whole genome methyl-seq (WGMS) allows assessment of DMRs across the entire genome and thus allows unbiased screening for changes in DNAm. To date, there are no whole-genome methylation studies that have investigated developmental exposure to air pollution in relation to the propensity for asthma.

In this study, a murine model of AAD is used with and without developmental exposure to PM_2.5_ to understand the impact of PM_2.5_ exposure during development on asthma outcomes, gene expression, and epigenomic correlates of these changes. It reveals that maternal exposure to PM_2.5_ is associated with profound changes in the transcriptomic response to OVA and identifies a potential role for widespread changes in the epigenome in this response.

## Results

### Maternal PM_2.5_ exposure increased AHR but did not alter pulmonary immune inflammation in adult progeny

**Fig. 1:**
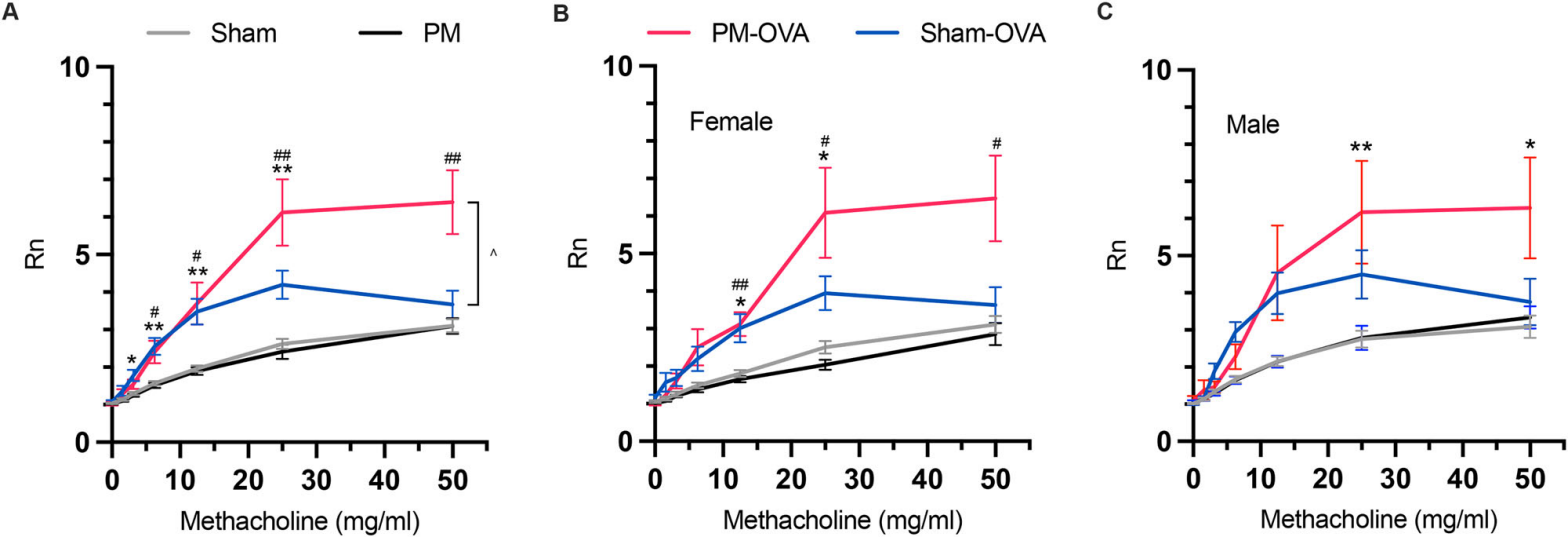
Lung function outcomes in response to developmental PM_2.5_ exposure and OVA challenge. A 13-week-old offspring across four groups were measured for airway hyperreactivity (AHR) in response to an increasing dose of methacholine using FlexiVent output of Newtonian resistance of the airways (Rn) for **A** both sexes combined (*n* = 21-26 per treatment), **B** female only (*n* = 11–14 per treatment), and **C** male only (*n* = 10–13 per treatment); data are presented as mean ± SEM. Univariate analysis of variance was performed + Tukey’s post-hoc test for multiple comparisons; *=*p* < 0.05 compared to Sham v Sham-OVA, **=*p* < 0.01 Sham v Sham-OVA, #=*p* < 0.05 PM v PM-OVA, ##=*p* < 0.01 PM v PM-OVA, and ^=*p* < 0.05.

A well-defined murine asthma model^32^ was used to evaluate the effect of air pollution exposure during the developmental window on subsequent AAD. Offspring from ambient air or PM_2.5_ exposed dams were challenged with OVA to induce AAD and lung function was assessed. Methacholine challenge showed OVA exposure caused significant dose-dependent methacholine-induced AHR (Fig. 1). Importantly, this increase was significantly higher in the PM-OVA group than in the control Sham-OVA group at high concentrations of methacholine (≥25 mg/ml). The analysis considered the effects of the covariates of litter size, litter sex skew (represented as a proportion of males in one litter), unique Dam ID, and sex on the major factors. Only the sex of the pups was a significant covariate affecting AHR, with the major effect being that females had a greater response to OVA at the highest methacholine dose (Fig. 1a–c).

**Fig. 2:**
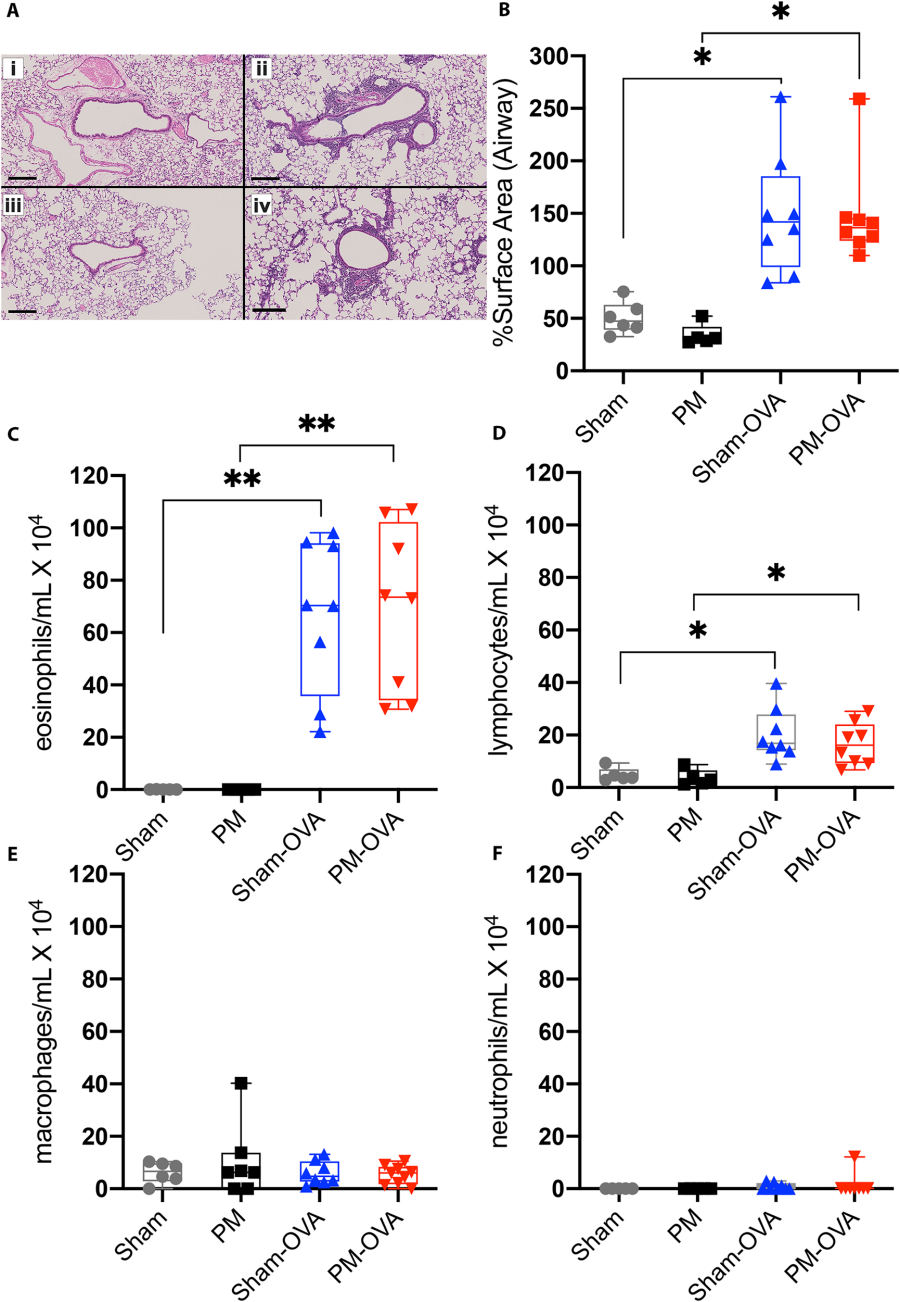
Inflammation outcomes in response to developmental PM_2.5_ exposure and OVA challenge. Representative histological images **A** of H&E stained lung sections (black bar = 200 μm) from 13-week-old female (i) Sham, (ii) Sham-OVA, (iii) PM, and (iv) PM-OVA (*n* = 5–8 per treatment) offspring, quantified for peribronchiolar inflammation **B** presented as median ± range and analysed with Kruskal–Wallis test + Dunn’s multiple comparison test; *=*p* < 0.05; **=*p* < 0.01. Post-lung function testing BALF cellularity **C**–**F** assessed via differential cell counts (*n* = 5–9 per treatment). Data are presented as median ± range and analysed with a mixed-effects model with the Geisser–Greenhouse correction + Tukey’s post-hoc test for multiple comparisons; *=*p* < 0.05; **=*p* < 0.01 (**C**).

Histological quantitative scoring of peribronchiolar inflammation showed an increase in Sham-OVA and PM-OVA compared to their respective controls but no difference between PM_2.5_ and Sham (Fig. 2a, b). BAL immune cell counts (Fig. 2c–f) showed OVA challenge significantly augmented pulmonary eosinophil and lymphocyte infiltration in Sham-OVA and PM-OVA compared to the respective saline-treated controls. Macrophage and neutrophil infiltration were not affected by the OVA challenge. There was no significant effect of maternal PM_2.5_ treatment across all leucocyte types assessed.

These results confirm that murine AAD in response to OVA challenge is associated with gross pulmonary inflammatory processes. However, the similarity in the inflammatory process in both Sham-OVA and PM-OVA offspring does not provide support for differences in gross inflammatory processes as a cause for the difference in AHR between these treatment groups. The sex effect is of interest and warrants further analysis, but to simplify this analysis, we contained further analyses of female offspring. To obtain a finer-grained picture of the difference in the respiratory response induced by maternal PM_2.5_ exposure we next assessed the transcriptome of lung tissue.

### OVA challenge altered the transcriptome of Sham offspring to a greater extent than PM offspring

RNA from whole lung tissue was extracted for RNA-Seq analysis. We performed cellular deconvolution (Supplementary Methods) of each replicate to determine if maternal treatment with PM_2.5_ changed the cellular response to the OVA challenge. The results of this analysis (Supplementary Fig. 2) are consistent with an OVA challenge inducing a dendritic cell-mediated Type II immune response. There was no difference in the cellular distribution profile between Sham and PM_2.5_ maternal treatment, with or without OVA challenge. The results of the deconvolution analysis are consistent with the histological analyses and taken together, the results do not provide support for the role of changes in nature or extent of the inflammatory process in response to OVA challenge being the primary effector of the altered pulmonary response in PM offspring.

**Fig. 3:**
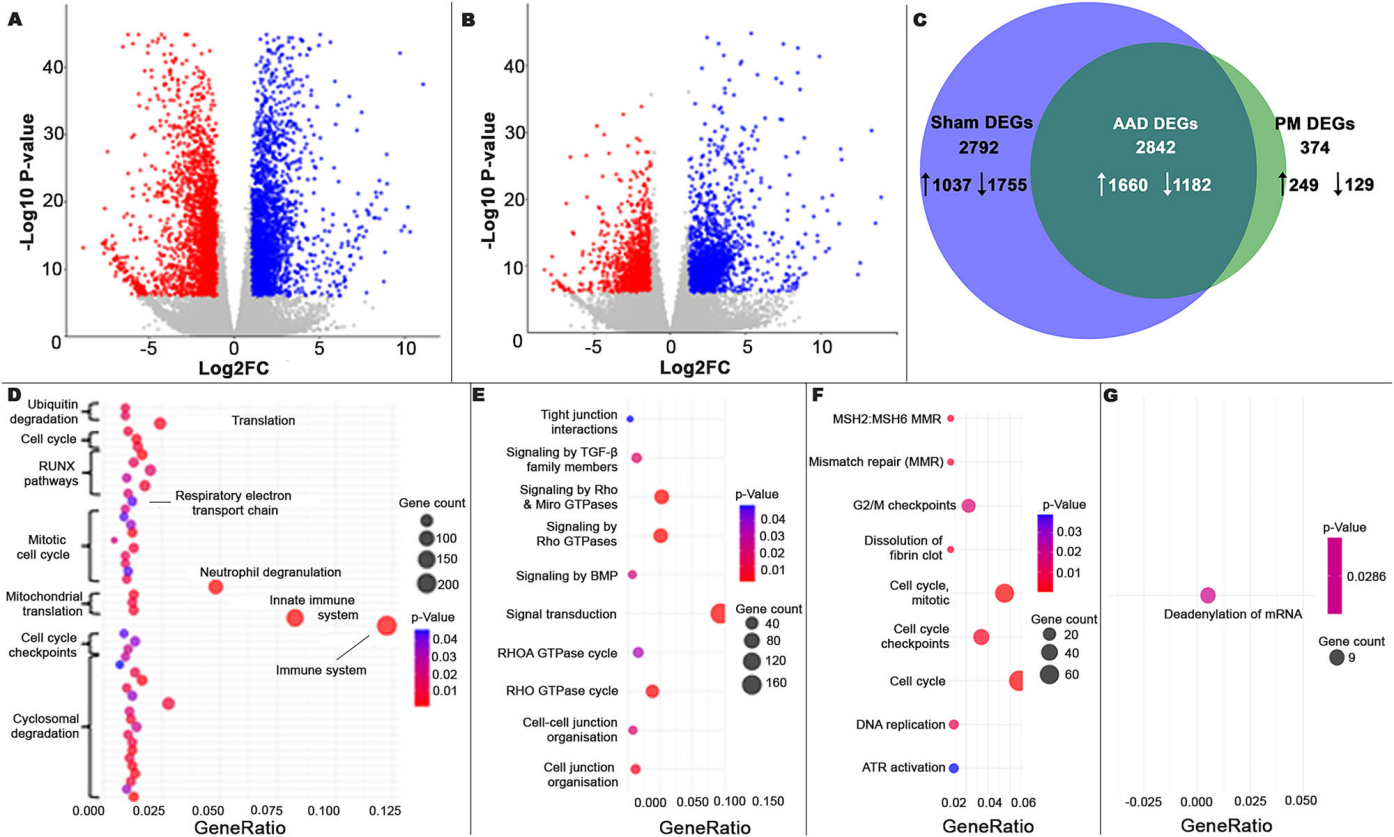
RNA-Seq performed on post-lung function tested 13-week-old female lung homogenates (*n* = 4–7 per treatment group). OVA treatment significantly induces differentially expressed regions (DEGs; *p* < 0.000001; log2FC > |1| in both Sham (**A**) and PM2.5 (**B**) offspring. Significant DEGs are denoted by colour (red = log2FC > 1, blue = log2FC < −1). **C** Set operations analysis showed how many of those genes were shared (AAD DEGs; dark green), unique to Sham (Sham; blue), and unique to PM_2.5_ (PM_2.5_; light green) offspring. The chi-squared analysis found that maternal insult significantly altered DEG expression patterns (*p* < 0.0001, d*f* = 2). **D**–**G** Bubble plots demonstrating DEGs enriched in key Reactome pathways (*p* < 0.05) for DEGs up (**D**) and downregulated (**E**) in AAD and those up (**F**) and downregulated (**G**) in Sham.

We showed that the OVA challenge caused a large transcriptomic response in both Sham-OVA (Fig. 3a) and PM-OVA (Fig. 3b) offspring compared to their respective saline controls. It was noteworthy, however, that the number of DEGs was greater in Sham-OVA than PM-OVA (Fig. 3c). Both the histological and cellular deconvolution analyses showed that a marked inflammatory process occurred in response to OVA challenge, so our analysis does not allow us to directly determine how much of the transcriptomic response to OVA was due to changes in function of pulmonary tissues compared to a measure of the transcriptomics of the inflammatory cells. Importantly though, cellular deconvolution analysis did not distinguish between Sham-OVA and PM-OVA treatment groups (Supplementary Fig. 2). This allows us to infer that the difference in the transcriptomic response in the PM-OVA treatment group was not primarily a consequence of changes in cellular allocation in the lung.

To analyse the impact of maternal PM_2.5_ treatment on the transcriptome in AAD, the OVA-induced DEGs from each group were compared using set operations analysis to classify them into different categories: (i) DEGs occurring in response to OVA challenge in both Sham and PM_2.5_ offspring (henceforth referred to as ‘AAD DEGs’), (ii) DEGs occurring in response to OVA challenge in Sham offspring only (henceforth referred to as ‘Sham DEGs’), and (iii) DEGs occurring in response to OVA challenge in PM_2.5_ offspring only (henceforth referred to as ‘PM_2.5_ DEGs’).

Applying this categorical separation of genes duplicated across treatment groups (AAD DEGs) and those unique to maternal treatment (Sham DEGs & PM_2.5_ DEGS) highlighted an effect of maternal treatment on differences in the differential expression of genes in response to AAD induction. We identified 2842 AAD DEGs (1660 upregulated and 1182 downregulated), 2792 Sham DEGs (1037 upregulated and 1755 downregulated), and only 374 PM_2.5_ DEGs (249 upregulated, 129 downregulated) (Fig. 3c). Chi-squared analysis showed a significantly greater proportion of Sham DEGs were downregulated, whilst a greater proportion from PM_2.5_ DEGs were upregulated.

Gene Set Enrichment Analysis (GSEA) using the Reactome key showed that the upregulated AAD DEGs were enriched for a range of pathways including those related to the immune system, synthesis of DNA, and regulation of the mitotic cycle (Fig. 3d); whilst those downregulated were enriched in cell signalling and cell-cell junction organisation pathways (Fig. 3e). Upregulated Sham DEGs were enriched in cell cycle and DNA mismatch repair pathways (Fig. 3f), whilst those downregulated were only enriched in the deadenylation of mRNA pathway (3 g). GSEA under the Reactome biological pathway of the 374 PM_2.5_ DEGs gave no results.

GSEA across Gene Ontology’s (GO) ‘molecular function’ aspect (Supplementary Table 1.1) showed the upregulated AAD DEGs to be enriched for protein and nucleotide binding pathways, whilst those Sham DEGs downregulated were enriched in many protein and nucleotide binding pathways with notable enrichment for SMAD and TGFβR pathways. By contrast, upregulated PM_2.5_ DEGs (Supplementary Table 1.2) showed no results across any GO aspect, and those downregulated were enriched in protein binding pathways. It is concluded that a major consequence of maternal PM_2.5_ exposure was a suppressed transcriptomic response to OVA challenge in several important key pathways. To assess the extent to which this altered transcriptome may be mediated by underlying changes in the epigenome we next investigated changes in the DNA methylome.

### Maternal PM_2.5_ exposure primed for a distinctive pattern of lower DNA methylation across the genome in response to OVA challenge

WGMS was performed to gain an understanding of the effect of maternal PM_2.5_ exposure across development on DNAm in the offspring’s lung and to assess how this changed after induction of AAD. A sliding window analysis that binned cytosines into 100 CpGs per window resulted in 420,017 probes across the entire genome. Of these, 245,315 windows overlapped annotated genes. Principal component analysis (PCA) of treatment groups showed a significant effect of AAD induction with OVA challenge (PC1 = 87%; Supplementary Fig. 3). Interestingly, when assessing along PC2 (6%) we report that Sham-OVA clusters with PM and PM-OVA clusters with Sham (Supplementary Fig. 3). Our results further showed there was no significant difference in global DNAm pattern between replicates in each treatment group (Supplementary Fig. 4). In the absence of baseline differences between Sham and PM, we posit that PC2 identified in our PCA analysis points to an interaction effect between maternal PM_2.5_ exposure and OVA challenge.

**Fig. 4:**
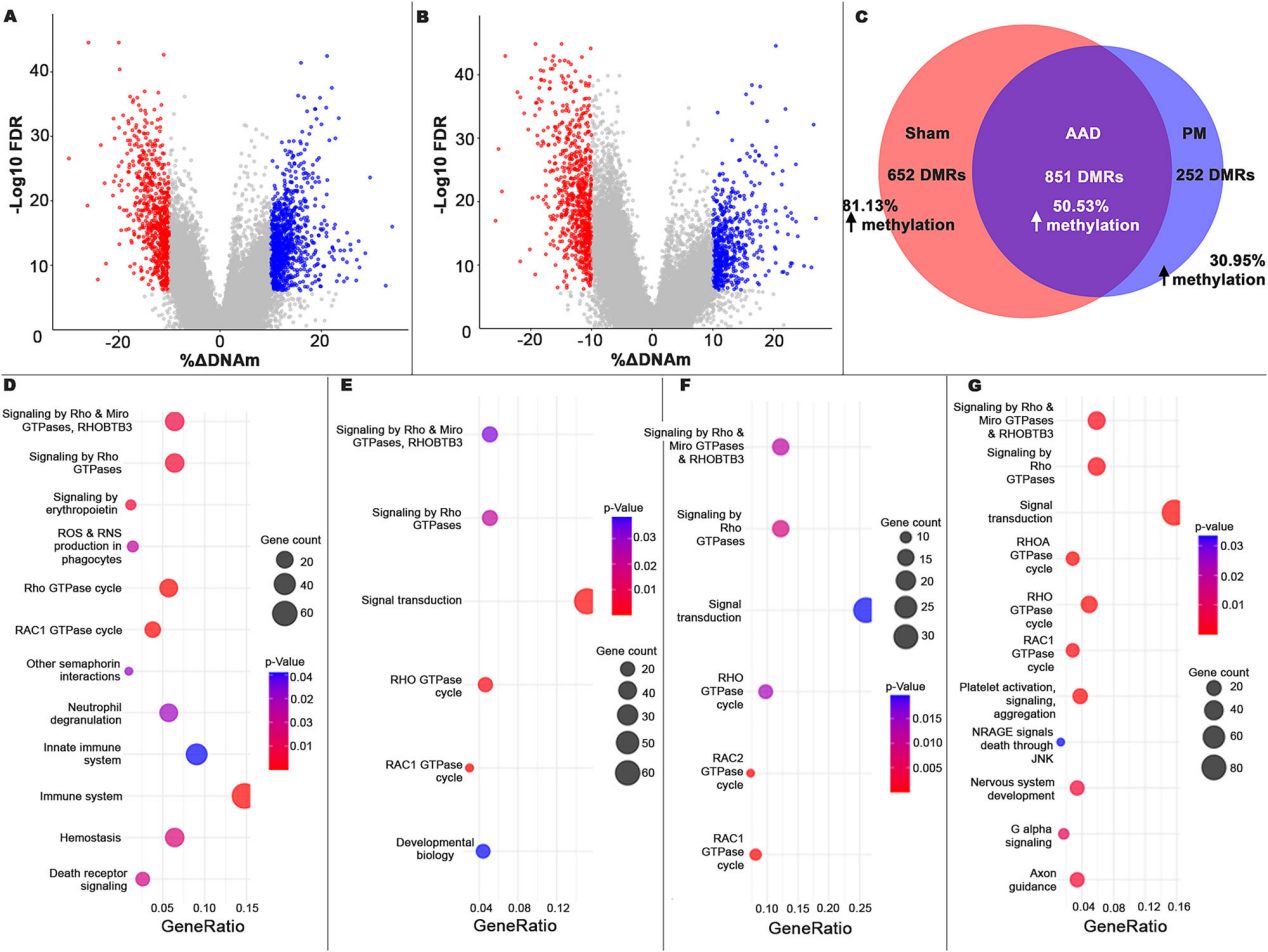
WGMS performed on post-lung function tested 13-week-old female lung homogenates from each group (*n* = 7–8 per treatment group). OVA treatment significantly induces differentially methylated regions (DMRs; assessed by EdgeR w/ threshold: *p* < 0.000001, ∆DNAm > |10%|) in Sham (**A**) and PM_2.5_ (**B**) offspring. Significant DMRs denoted in colour (red = decrease in DNAm; blue = increase in DNAm). **C** Set operations analysis showed how many of those DMRs were shared (AAD DMRs; dark purple), unique to Sham (Sham; red), and unique to PM_2.5_ (PM_2.5_; light blue) offspring. The chi-squared analysis found that maternal insult significantly altered DEG expression patterns (*p* < 0.0001, d*f *= 2). **D**–**G** Bubble plots demonstrating DMRs enriched in key Reactome pathways (*p* < 0.05) DMRs up (**D**) and downregulated (**E**) in AAD and those up (**F**) and downregulated (**G**) in Sham.

When analysing for differentially methylated regions (DMRs) that overlapped genes (245,315 windows), the OVA challenge was shown to induce many DMRs in both Sham and PM_2.5_ offspring (Fig. 4a, b), with more DMRs in Sham. Since cellular deconvolution showed that OVA treatment was associated with an inflammatory process, but that Sham and PM groups were not distinguishable, we therefore can’t infer whether the DMRs associated with OVA challenge were a consequence of changed cellular function or cell allocation, but we can infer that any effect of PM_2.5_ on DMRs was not primarily due to changes in cellular allocation. Although there were no significant DMRs when comparing Sham with PM_2.5_ both at baseline (Supplementary Fig. 5a) and following OVA challenge (Supplementary Fig. 5b), there was not complete concordance between the Sham and PM_2.5_ offspring in the DMRs induced by OVA challenge. Similar to our DEG analysis, DMRs were split into three different categories: (i) DMRs occurring in response to OVA in both Sham and PM_2.5_ offspring (AAD DMRs), (ii) DMRs only occurring in response to OVA in Sham offspring only (Sham DMRs), and (iii) DMRs only occurring in response to OVA in PM_2.5_ offspring only (PM_2.5_ DMRs). Overall, we detected 851 AAD DMRs, 652 Sham DMRs, and a smaller 252 PM_2.5_ DMRs (Fig. 4c). The AAD DMRs showed an approximately equal split between those that resulted from shifts towards an increase or decrease in DNAm (50.5% showed ≥10% increase %DNAm score). By contrast, the majority of Sham DMRs showed an increase in DNAm (81.1% showed ≥10% increase %DNAm score), whilst the majority of PM_2.5_ DMRs showed a decrease in DNAm (69.1% showed ≥10% decrease in %DNAm score).

### Maternal PM_2.5_ exposure attenuated DNAm changes in developmental, transferase activity, nucleotide binding, and GTPase signalling pathways

Many DMRs that overlapped annotated genes were identified, and in some instances, multiple DMRs overlapped with a single gene. As such, we deduplicated our DMR lists to compile unbiased lists and subjected them to GSEA. We henceforth refer to our deduplicated list of DMRs overlapping genes as differentially methylated genes (DMGs). GSEA against Reactome showed the AAD DMGs were predominantly enriched in pathways affecting cell signalling (Fig. 4d, e). Whilst Sham DMGs were enriched in cell signalling, signal transduction, and developmental pathways (Fig. 4f, g), PM_2.5_ DMGs did not show enrichment in any pathways. We furthered this analysis by considering GSEA against the GO molecular function aspect.

Sham DMGs with shifts to higher DNAm were enriched in numerous pathways related to protein binding and signalling, with ‘transferase activity’ and ‘nucleotide binding’ pathways only occurring in this treatment subset (Supplementary Table 1.3). Sham DMGs showing lower DNAm were enriched in pathways related to protein binding and GTPase signalling. PM_2.5_ DMGs showing higher DNAm were enriched in pathways related to binding, whilst those that showed higher DNAm were enriched in numerous protein binding, nucleotide binding, and transcription pathways (Supplementary Table 1.4). Next, the proportion of DMGs that corresponded with DEGs in each treatment group was assessed.

### A preponderance of Sham DMGs, but not PM_2.5_ DMGs, were associated with altered transcription

**Fig. 5:**
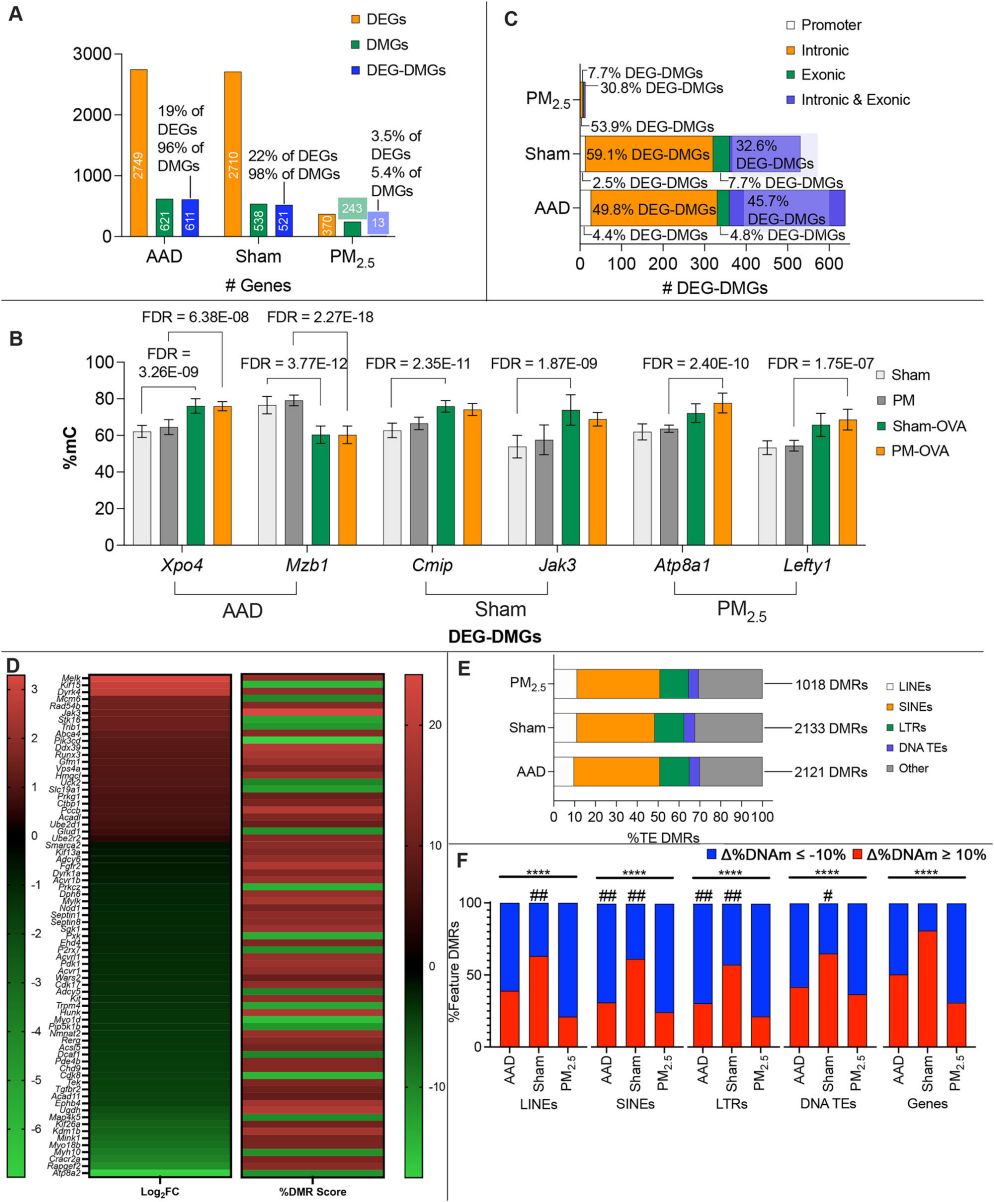
DEG-DMGs across genomic features. **A** Number of annotated genes represented by DEG-DMGs (blue) identified using Key Set Operations analysis of DMGs vs DEGs, DMGs (green), and DEGs (orange) demonstrating a putative relationship between differential gene expression and methylation **B** intermediate methylation values of representative DEG-DMG genes showing high differential expression and methylation represented as %methylation (methyl calls per 100 CpGs). **C** Breakdown of which nuclear features (promoter = white; introns = orange; exons = green; introns & exons = blue) DEG-DMGs overlap. **D** Ranking log_2_FC with respective %DMR score for DEG-DMRs enriched for ‘nucleotide binding’ found that there was no clear relationship between DEG up or downregulation with higher or lower DNA methylation. **E** Determining which DMRs overlapped with transposon classes showed that there was no shift in DMR representation across transposon classes induced by treatment. **F** %DNAm decrease (blue) and increase (red) across all classes of TE DMRs alongside Gene DMRs. The chi-squared analysis found that shifts to higher or lower DNA methylation patterns were significantly different across treatments within each nuclear feature (****=*p* < 0.0001), and when comparing TE order DMRs with Gene DMRs within respective treatment groups (#=*p* < 0.05, ##=*p* < 0.01 when compared with Gene DMR in the respective treatment group).

Assessing which DEGs overlapped with DMGs (DEG-DMGs) found 611 AAD DEG-DMGs, 521 Sham DEG-DMGs, and only 13 PM_2.5_ DEG-DMGs. Analysis of the proportional representation of total DMGs within the DEG-DMGs detected found a gross majority of AAD and Sham DMGs were associated with DEGs, whilst only a small proportion of PM_2.5_ DMGs were associated with DEGs (Fig. 5a). Analysis of the proportional representation of overall DEGs within the DEG-DMGs for each respective gene set (AAD, Sham, PM_2,5_) found DMGs only accounted for 22% and 19% of DEGs in the AAD and Sham cohorts, respectively. Whilst PM_2.5_ DEG-DMGs accounted for 3.5% of PM_2.5_ DEGs. Figure 5c shows exemplar DEG-DMGs with both higher differential gene expression and DNAm.

The results show that a subset of differentially expressed genes was associated with a measurable change in DNAm level across the gene body. However, almost all gene bodies with differential DNAm were associated with altered gene expression in Sham offspring. By contrast, in genes unique to PM_2.5_ offspring, only a small proportion showed differential DNAm were associated with differential gene expression.

Given that DNAm at different nuclear functional regions modulates gene expression differently^56–59^, we sought to determine if there was a broad pattern of transcriptional direction in our DEG-DMGs. To investigate this, a subset of DEG-DMGs enriched for GO molecular function classification of ‘nucleotide binding’ (GO:0000166) were identified and ranked by log2 fold change (Log2FC) of gene expression against their respective % shift in DMR score (Fig. 5d). This showed there was no obvious direct relationship between up or downregulation of transcription and ≥10% shift in %DNAm, suggesting the relationship between altered DNAm and transcription is complex and warrants investigation of the genomic location of DNAm shifts to highlight regions of interest. Our findings show that maternal PM_2.5_ exposure changes the nature of the association between gene DNAm levels and transcriptional activity of genes and warrants further investigation of this.

### OVA-induced DMRs predominantly occur in intronic regions

Genes are a composite of several functionally definable regions (such as gene promoters, introns, and exons) so we next assessed whether any of these regions were preferential targets for differential DNAm. This analysis was in the subset of genes that showed differential expression. The large majority of DMRs overlapped intronic regions, with progressively fewer DMRs overlapping exonic, and promoter regions, respectively (Fig. 5b). Considering that intronic regions have relatively high concentrations of transposable elements (TEs) in mammals^60,61^—which are common targets of DNAm^62^—we next aimed to identify which TE classes were a target for differential DNAm in AAD.

We investigated the number of DMRs that overlapped common orders within Class I (long interspersed nuclear elements (LINEs), short interspersed nuclear elements (SINEs), long terminal repeats (LTRs)) and Class II (DNA) TEs (Fig. 5e). Similar to the gene DNAm results, of the three treatment groups, PM_2.5_ had the lowest number of differentially methylated TEs (1018 DMRs). Across all treatment comparisons SINEs represented the largest group of TEs with DMRs (Fig. 5e). Maternal PM_2.5_ treatment did not significantly alter the proportions of each order of TEs showing global DNAm changes across TEs, demonstrating no skew across orders caused by maternal treatment (Fig. 5e). Those TE’s with DMRs occurring in AAD (Fig. 5f) showed a shift towards decreased %DNAm across all TE classes, whilst Sham DMRs overlapping TEs predominantly showed higher %DNAm, and those PM_2.5_ DMRs overlapping TEs predominantly showed lower %DNAm. However, compared to genes as a whole, each order of TEs assessed (Fig. 5f) showed a skew towards lower %DNAm across all treatment groups, with significant shifts across all TE orders in Sham, and SINEs and LTRs in AAD.

### The endothelial cell was a cell-type signature of DEG-DMG gene expression

**Fig. 6:**
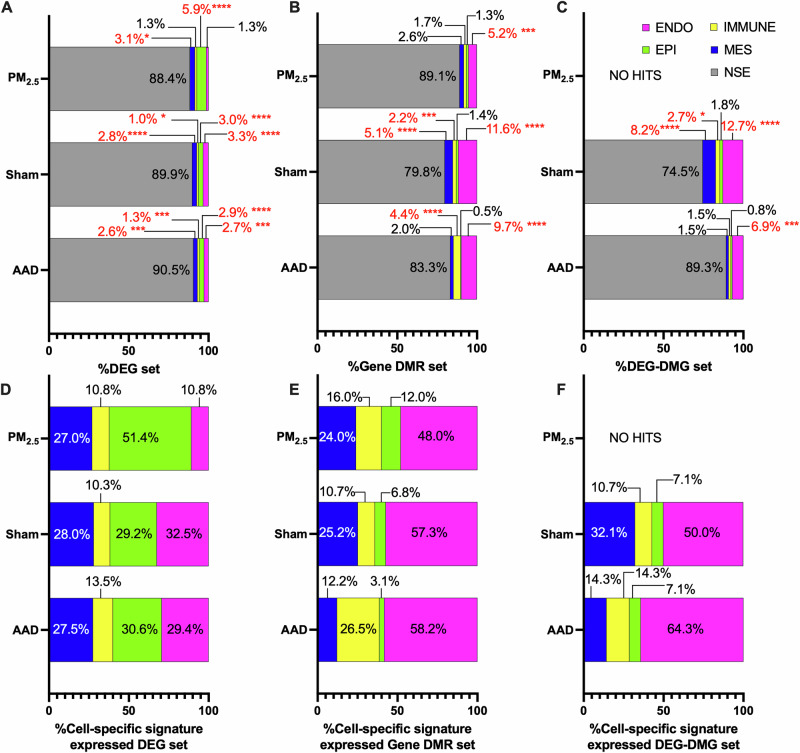
Lung gene expression atlas. Enrichment performed differential expression *t*-test *p*-value cutoff <0.05, with non-selective expression defined by cross-cell comparison differential expression test *p*-value ≥ 0.05, for DEGs (**A**, **D**), DMRs (**B**, **E**), and DEG-DMGs (**C**, **F**) (pink = endothelial cells; green = epithelial cells; yellow = immune cells; blue = mesenchymal cells; grey = genes not selectively expressed from a single cell type; red font highlights statistically significant results: *=*p* < 0.05, *=*p* < 0.01, ***=*p* < 0.001, ****=*p* < 0.0001).

Assessing cell-type signature gene expression profiles of DEGs across all three groups showed AAD and Sham groups were similar, with significant cell-type signature gene expression from endothelial, epithelial, immune, and mesenchymal cells with the lowest proportion of cell-type signature genes being attributed to immune cells (Fig. 6a, d). These cell-type signature results are further emphasised by our transcriptomic cellular deconvolution results, wherein no detectable difference between Sham and PM-treated offspring in proportion to all detectable cell types with or without OVA challenge was shown. PM_2.5_ only had significant cell-type signature gene expression from mesenchymal and epithelial cells, with epithelial cells representing the highest proportion of cell-type signature expressed genes. See Supplementary Table 2 for a list of cell-type specific gene drivers.

Assessing DMRs, AAD showed significant cell-type signature gene expression from endothelial and immune cells, with endothelial cells representing the highest proportion (Fig. 6b, e). Sham showed significant cell-type signature gene expression from endothelial, mesenchymal and immune cells, whilst PM_2.5_ only showed cell-type signature gene expression from endothelial cells. Across all cohorts, the greatest proportion of DMRs were associated with cell-type signature genes expressed from endothelial cells. The lowest rate of non-selective expression (NSE) was observed in Sham and the highest in PM_2.5_. See Supplementary Table 2 for a list of cell-type specific gene drivers.

Assessing DEG-DMGs, AAD DEG-DMG genes only showed significant cell-type signature expression from endothelial cells, whilst Sham DEG-DMG genes showed significant cell-type signature expression from endothelial, mesenchyma and immune cells (Fig. 6c, f). As with DMRs, the greatest proportion of DEG-DMG cell-type signature expression was driven by endothelial cells. The greatest proportion of NSE was observed in AAD, followed by Sham. Due to the low number of DEG-DMGs in the PM_2.5_ group, no cell-type signature gene expression hits were detected. See Supplementary Table 2 for a list of cell-type specific gene drivers.

## Discussion

This study models murine AAD using an allergic challenge to manifest features of human asthma, such as increased eosinophilic inflammation and AHR. This was used to assess the effects of maternal exposure to air pollution throughout the developmental window of her progeny on their airway responsiveness as adults. Our findings confirm^32,63^ that maternal air pollution particulate exposure through development led to altered lung function in the offspring and shows, for the first time, that exposure to PM_2.5_ during the developmental period results in increased airway Newtonian Resistance (Rn) in response to methacholine challenge in adult offspring.

Histological and cellular deconvolution analyses showed that the heightened AHR response in PM offspring was not associated with gross changes in the inflammatory profile of the lung but was associated with marked changes in the organ’s transcriptomic profile. The induction of AAD generated large changes in the pattern of gene expression, a major proportion of which was shared between both control (Sham) and PM_2.5_ treatment groups. There was shared enrichment in gene expression pathways related to the immune system, DNA synthesis, mitotic cycle, cell signalling and cell–cell junction organisation. The study design does not allow us to assign these changes to a particular cell type but would include the effects of the introduction of immune cell types into the lung. Importantly, however, there was a significant portion of the transcriptomic response that was unique to the form of maternal exposure, and this occurred independently of any detectable changes in cellular composition. Most notably, developmental PM_2.5_ exposure abrogated a significant portion of the transcriptomic response observed in Sham offspring, thereby reducing the changed expression of genes enriched for numerous important biological pathways such as cell cycle, DNA mismatch repair, and deadenylation of mRNA. It is concluded that maternal exposure to air pollution particulates caused a markedly diminished transcriptomic response by her progeny to respiratory allergic challenges. This difference in both lung function and gene expression in the offspring of dams exposed to air pollution suggests that the treatment resulted in an underlying change in the biology of the offspring that serves as a memory of its conditions of conception and development. Given that the increased AHR in PM_2.5_ offspring was associated with fewer changes in the expression of a subset of genes, this may infer that the affected genes normally have roles in mitigating the response to allergic challenges. This question warrants direct investigation.

DNAm is a pervasive epigenetic mechanism involved in regulating chromatin structure and patterns of gene expression. It has been implicated as an important mechanism in some models of the developmental origins of disease^35,36,64^. The window of time between ceasing exposure to PM_2.5_ (PND 21) and collection of samples (PND 91) in this study demonstrates that developmental PM_2.5_ exposure induced genome-wide aberrations in gene expression and methylation that continue into adulthood. However, the whole-genome screening of enzymatically converted^65^ DNAm of lung tissue not subjected to allergic challenge showed no difference between control and air-pollution particulate exposed offspring. This infers that a change in the DNAm profile is not a likely candidate for the direct molecular memory of environmental pollution during development. By contrast, differential DNAm did mirror many of the changes in pulmonary differential gene expression following allergic challenge. As with gene expression, AAD induced many changes in DNAm that were shared by both control and pollutant-exposed groups. However, many differentially methylated regions occurring in Sham-treated offspring did not occur in offspring subjected to developmental PM_2.5_ exposure. This difference occurred across genes enriched in numerous important biological pathways such as cell signalling and developmental biology.

It is noteworthy that the differential DNAm was not due to a small number of sites that show large quantitative changes in the level of DNAm, rather it was predominantly due to a relatively lower level of differential DNAm across a large number of sites. Such intermediate shifts in DNAm are reported to be strongly enriched in genes, enhancer chromatin states and evolutionarily conserved regions^46,66,67^, associated with intermediate levels of active histone modifications^46^, and pathological *KRAS* mutation in precancerous colorectal lesions^68^. Furthermore, intermediate DNAm signatures have shown to be consistent across tissue, peripheral blood and purified cell types^46,66^ suggesting that intermediate DNAm may be a stable state of DNAm variability across cells of the same type within a population rather than an artefact of differential DNAm across cell types. The relationship between intermediate levels of differential DNAm and altered gene expression is of great interest and warrants further investigation.

Our finding that PM_2.5_ treatment of dams induced a unique subset of differentially methylated genes, with a skew towards decreased DNAm, is of interest when taken in the context of recent work analysing human asthmatic methylome array datasets. These show that differential DNAm in asthma predominantly results in hypomethylation in patients^69–71^.

Clinical samples aligned with spatiotemporally determined levels of PM_2.5_ exposure averaged over the first and second trimesters are associated with increased salivary CpG methylation residing within inflammatory pathway gene *TMEM184*^50^ in offspring and differentially methylated CpG sites and regions in peripheral whole blood, overlapping annotated genes enriched in numerous key biological pathways, including positive regulation of blood vessel diameter, phospholipase activity and inflammatory mediatory regulation of TRP channels^51^, which shows that our results are reflective of DNAm changes associated with air pollution exposure in clinical cohorts. However, our findings are more informative in that we control the direct PM_2.5_ exposure and use whole-genome DNAm sequencing for unbiased analysis.

Analysis of cell-specific signatures of gene expression point to lung endothelial cells being an important target for the effects of air pollution in offspring and warrant further investigation of this cell type. This result may be consistent with findings that endothelial dysfunction is associated with asthma in humans^72^. In assessing the relationship between changes in gene methylation and expression, we show that over 96% of differentially methylated genes were also differentially expressed in those genes shared amongst AAD and those unique to Sham developmental exposure. However, developmental exposure to PM_2.5_ altered this dynamic, with only 5.4% of differentially methylated genes unique to this group being differentially expressed. This demonstrates for the first time a shift in the regulatory relationship between pulmonary gene DNAm and expression due to developmental exposure to PM_2.5._

Of those genes that were differentially methylated and expressed, no strong relationship between higher or lower gene methylation and expression levels was observed, suggesting a complex relationship between developmental PM_2.5_ induced DNAm patterns and gene expression. The most well-understood mechanism through which DNAm alters gene expression includes instances of DNA hypermethylation across CpG-rich TSSs, which silence genes by modulating access of transcription factors and DNA binding proteins to promoter regions^56,58^. However, instances of DNA hypomethylation leading to gene repression have been reported^57^, and in some instances, hypermethylation of the promoter region may lead to gene activation in normal tissue and suppression in disease^59^. The complex dynamics between DNAm and gene expression can be attributed to the many cis-regulatory ways DNAm interacts with chromatin, transcription factors and chromatin-modifying proteins. For example, loss of DNAm coupled with repressive chromatin marks has been shown to silence genes^57^. Whilst gain of DNAm can impede spliceosome mediating factors to induce alternative splicing^44^, silence enhancers^41^ and prevent spurious entry of RNA Pol II at cryptic intergenic promoters^43^ to maintain gene expression. Other factors that may affect DNAm include the abundance and activity of DNAm modifying enzymes (DNMT1, DNMT3A, DNMT3B, TET1, TET2 and TET3) and their protein partners (DNMT3L and UHRF1). Future analysis of the effects of air pollution particulates on these epigenetic modifiers is warranted.

Upon assessing feature-specific differential DNAm across promoter regions, introns and exons, a greater proportion of concurrent differential DNAm and gene expression was observed in intronic regions. Given that DNAm is most pronounced in exonic regions across the mammalian genome^73,74^, these findings are of interest as they may demonstrate the relative stability of exonic DNAm while pointing to a susceptibility of the fidelity of intronic DNAm as a consequence of developmental environmental insults. Intronic cytosine DNAm in mammals has been shown to affect gene expression in cis^75^ by acting as internal promoters, enhancers and stabilising mRNA^76^.

Mammalian intronic regions are abundant with TEs^60,61^. TEs are widespread throughout the genome and are generally hypermethylated, ensuring their transcriptional silence^62^. TEs have been shown to undergo rapid DNA demethylation and subsequent increased expression during the large epigenetic reprogramming events that occur during pre-implantation development and gametogenesis^77,78^. DNA demethylation dynamics affect TE orders differently during epigenetic reprogramming events. For example, LTR TE families, known as intracisternal A particle (IAP) elements and endogenous retroviruses (ERVs), are most resistant to demethylation, whilst the LINE TE family, known as LINE1s, and SINEs, are most susceptible to DNAm reprogramming during primordial germ cell development^79^. Clinical studies show an association between prenatal environmental insults—such as prenatal tobacco smoke^80^ or spatiotemporally estimated exposure to air pollution^52,54^—and changes in TE DNAm. For example, kindergarten-aged children exposed to prenatal tobacco smoke had lower peripheral blood DNAm of SINE family member, AluYb8, at the global level and LINE elements only when associated with a GSTM1null gene variant^80^. It should be noted that the effects of prenatal environmental exposure and TE DNAm are complex and may vary with subject age, specific air pollutant exposure and period of exposure. For example, prenatal air pollutant associations with DNAm measured in matched cord blood and peripheral blood collected at 1 year of age showed that prenatal NO_2_ exposure was associated with higher L1 LINE element DNAm in cord blood and lower L1 LINE element DNAm at 1 year^54^; whilst another study showed air pollutant exposed offspring showed lower LINE1 element DNAm if exposed to PM_10_ and O_3_ in the first trimester and higher LINE1 element DNAm if exposed to O_3_ in the third trimester^52^. Our observation that direct developmental exposure to PM_2.5_ caused differential methylation across LINEs, SINEs, LTRs and DNA TEs, with no order affected to a greater magnitude than another, builds on this body of work by using a controlled dose of exposure and unbiased DNAm analysis to shed light on the effect of a specific component of air pollution on TE DNAm. A broad shift towards lower DNAm across all TE orders in Sham and PM_2.5_ offspring was observed. TE hypomethylation may promote TE expression^81,82^. Importantly, these hypomethylated TEs are reported in some instances to act as cryptic promoters^83^ or tissue-specific enhancer marks^84^ allowing for non-canonical regulation of gene expression. TE upregulation in human disease has been reviewed^85,86^ and may act through inducing genomic rearrangement, deleterious insertions and modulating epigenetic controls.

In the context of lung disease, possible roles for TEs expression are in the nascent stages of investigation. However, it has been shown that ‘LINE1’ elements play a role in lung diseases, wherein LINE1 activation has been shown to induce inflammatory processes in idiopathic pulmonary fibrosis(IPF)^87^ and asthmatics exposed to diesel exhaust showed differential DNAm of LINE1 and Alu elements compared to asthmatics exposed to filtered air^88^. Taken together, this evidence points to an interaction between air pollution exposure and changes in the DNAm of TEs and altered propensity for increased asthma. It should be noted that our short-read sequencing data sets do not offer the resolution necessary to capture all TE orders relative to differentially expressed genes and future studies should use long-read technology to elucidate the effects of shifts in TE DNAm on TE expression in the context of developmental PM_2.5_ exposure.

It is noteworthy that while this study revealed a strong association between altered DNAm and the differential gene expression of a wide spectrum of genes, for many genes that were differentially expressed we did not detect any change in DNAm across the gene or in its likely regulatory regions. This does not exclude the possibility that more distal changes in DNAm had a role in mediating these changes^89,90^. However, it may also infer roles for other epigenetic effectors in mediating the effects of air pollution particulates in the heightened pulmonary allergic reaction in this model. Indeed, roles for other epigenetic effectors, including histone methylation^91,92^ and acetylation^93–95^ marks, have been implicated in the severity of asthma, and a genome-wide association study identified significant histone modifying enzyme (KDM2A, KDM4C, HDAC4, HDAC7 and HDAC9) polymorphisms^96,97^ in asthma.

Asthma is a heterogeneous disease with numerous molecular endotypes^98^. We used an established OVA model to initiate AAD to elicit a Type II immune response, whilst house dust mite would be used to elicit an interferon-gamma (IFN-γ) mediated Type I and T_H_17 responses^98^. Each endotype is representative of a clinical population, thereby warranting future investigation of maternal insult on each endotype. House dust mite is an important target for future analysis, representing a real-world allergen within the clinical population. Another noteworthy limitation pertains to the collection and processing of PM_2.5_. We have noted that air pollution is a complex mixture of gases and particulate matter, with heterogeneity reflective of collection source^99^. We collected our PM_2.5_ from Hong Kong and normalised to Sydney, Australia levels to show the health effects of low air pollution on AAD and have characterised PM_2.5_ composition to report on organic carbon, elemental carbon and water-soluble inorganic ions. As such, we do not report on the gaseous components of air pollution, warranting further study into the effect of these components of air pollution on AAD.

This work reports important novel findings relevant to our understanding of the molecular mechanisms underlying increased asthma severity in adult offspring due to maternal exposure to air pollution particulates throughout development. This environmental insult throughout development led to a more severe asthmatic AHR outcome in adults and transcriptomic and whole-genome methylation sequencing showed that maternal exposure to air pollution was associated with widespread abrogation of DNAm and gene expression in several key biological pathways in their offspring. PM_2.5_ predominantly induced DNAm changes in introns and SINE elements, especially within genes. This change tended to skew towards lower DNAm. Many differentially methylated and expressed genes are selectively expressed by pulmonary endothelial cells potentially implicating this cell type as mediating this changed response. These findings are an important step towards understanding the sustained changes induced by developmental maternal exposure to an environmental insult known to underpin a propensity for increased asthma severity.

## Materials and methods

### PM_2.5_ preparation

PM_2.5_ were collected as previously described^32^. Briefly, in the summer, PM_2.5_ was collected from a busy roadside in Hong Kong using particulate matter (PM) samplers (URG-2000-30EH, 8 L/min) through a 47 mm Teflon (Pall Life Sciences, Ann Arbour, MI, USA) and (800 °C, 3 h) 47 mm quartz-fibre filters (Whatman, Clifton, NJ, USA). The PM was extracted from the filters with 90% ethanol and 5 min of sonication and dried overnight by freeze-drying. The contents of organic carbon and elemental carbon were analysed using a Desert Research Institute Model 2001 Thermal/Optical Carbon Analyser with the IMPROVE-A protocol. Water-soluble inorganic ions were determined by ion chromatography (Supplementary Fig. 1a). PM size was determined by dynamic light scattering (Microtrac252, Montgomeryville, PA, USA) (Supplementary Fig. 1b).

### Animal experiments

Animal experiments were approved by the Animal Care and Ethics Committee of the University of Technology Sydney (ETH18-3175). We have complied with all relevant ethical regulations for animal use. All protocols were performed according to the Australian National Health and Medical Research Council Guide for the Care and Use of Laboratory Animals. Female BALB/c mice (*Mus musculus*) (6 weeks, Animal Resources Centre, Perth, WA, Australia) were housed at 20 ± 2 °C and maintained on a 12 h light, 12 h dark cycle (lights on at 06:00 h) with *ad libitum* access to standard rodent chow and water; all animals were acclimatised for 1 week prior to the commencement of experimentation. Female BALB/c mice were divided into two groups, Sham (*n* = 15) and PM_2.5_ (*n* = 15) dams. The PM_2.5_ dams were exposed to PM_2.5_ standardised to Sydney, Australia levels^100,101^ (5 µg/day intranasal exposure) suspended in 40 µL saline (20 µL each naris) prior to mating for 6 weeks, during the entirety of gestation (~3 weeks), and during weaning (~3 weeks). The Sham dams were exposed to saline (0.9% NaCl; 40 µL in total, 20 µL each naris) for the same period. Our PM_2.5_ dam treatment schedule recapitulates maternal exposure to air pollution prior to mating, during gestation and through weaning. Offspring were never directly exposed to PM_2.5_, however they were exposed those components of PM_2.5_ that pollute lactation, as has been shown in real world studies^102^. As such, our model of real-world maternal exposure to the offspring is termed the “developmental window” of exposure.

### OVA-induced airway hyperresponsiveness

At 7–9 weeks, male and female offspring from 15 Sham and 15 PM_2.5_ dams were evenly allocated across groups ± OVA challenge, yielding four offspring treatment groups: (i) Sham (*n* = 22), (ii) Sham-OVA (*n* = 20), (iii) PM (*n* = 28), (iv) PM-OVA (*n* = 20). Based on our previously described asthma model^32^, we made minor adjustments to the established AAD model. Briefly, each male and female offspring were sensitised with OVA (100 µg, i.p.) adsorbed with 2 mg Al(OH)_3_ in sterile saline on days 0 and 14, followed by aerosolised (Aeronob Nebuliser) OVA (1% in sterile saline, 30 min) challenge on a postnatal day (PN) 19, 21, 23, 25 and 27. Saline control animals receive nebulised saline for the same duration. The lungs were collected the next day after the last OVA administration, with the left lung formalin fixed, and the right snap frozen.

### Lung function tests

FlexiVent (SCIREQ, Montreal, QC, Canada) apparatus was used to measure AHR in response to increasing doses of methacholine (Sigma-Alrich, St Louis, MO, USA) using the forced oscillation technique. Briefly, male and female offspring were anaesthetised (tribromoethanol, 250 mg/kg, Sigma-Aldrich, St Louis, MO, USA) and tracheostomised. Then, an 18-gauge polyethylene cannula was inserted. The cannula was connected to the flexiVent and ventilated at 200 breaths/min with a tidal volume of 10 mL/kg and a positive end-expiratory pressure of 3 cm H_2_O.

Lung function was performed at baseline and after increasing doses of methacholine (0, 1.6, 3.125, 6.25, 12.5, 25 and 50 mg/mL, Sigma-Alrich, St Louis, MO, USA) generated by an in-line nebuliser to measure airway reactivity. Two deep inspirations were performed before each dose to standardise volume history. The impedance of the respiratory system was fit to calculate Newtonian resistance (Rn, reflecting airway resistance). The results were the mean of the three consecutive peak measurements at each dose. Lung samples were humanely collected at this experimental endpoint.

### Bronchoalveolar lavage (BAL) fluid

Immediately after the lung function test, saline (0.5 mL, twice) was used to collect the BAL fluid, which was then centrifuged. The cell pellet was resuspended in 1 mL phosphate-buffered saline (PBS). The cell suspension was mixed with 4% Trypan blue (1:1, Life Technologies, Carlsbad, CA, USA). Total cells were then counted using a haemocytometer. Cytospin slides were then made (Thermofisher Scientific, Waltham, MA, USA). A differential cell count was performed by counting four random fields of view under a light microscope by an observer blinded to the treatment of the mice.

### Histology

Peribronchial inflammation of haematoxylin and eosin-stained sections were graded using a published method^103^. Quantitative scoring of peribronchial inflammation, controlling for airway lumen size, was carried out using ImageJ^104^ (National Institute of Health, Bethesda, MD, USA) with the Fiji^105^ plugin. Feature measurements were performed in frames containing one or more complete airways with no bronchial branching in the frame. Measurements from 3× airways from each replicate were averaged to produce the lumen area (LA) and the total circumference of peribronchial inflammation (SA) per replicate. Subtracting the LA from the SA allows for peribronchiolar inflammation expressed as a proportion of lumen size; calculated using [(SA − LA)/LA) * 100] to express as a percentage.

### Tissue homogenisation and nucleic acid extraction

A 10 mg portion of snap frozen right lower lobe lung from a 13-week-old female offspring was homogenised using the Precellys^TM^ Tissue Homogeniser (Bertin Technologies, IDF, France). Sample mRNA and gDNA were extracted using the AllPrep DNA/RNA/Protein Mini Kit (QIAGEN, Hilden, Germany) as per the manufacturer’s instructions. Quantification of mRNA and gDNA was carried out with a NanoDrop2000 (Themofisher, North Ryde, Australia).

### RNA-seq and data analysis

#### Library preparation and sequencing

Extracted RNA samples (*n*: Sham=7; PM = 7; Sham-OVA = 5; PM-OVA = 4) from female offspring underwent quality control (QC), library prep and RNA sequencing at the Ramaciotti Centre at UNSW Sydney. RNA libraries were sequenced as 100 bp single-end reads on Illumina NovaSeq 6000.

#### Data handling

Reads were trimmed with Trimmomatic (version 0.39), mapped to GRCm38 with Bowtie2 (version 2.5.1) and SAMtools (version 1.17), and log2-transformed reads per million were quantified and statistically analysed (DESeq) using SeqMonk (v1.48.1; Babraham Institute). See statistics methods for details on statistical analysis.

### Whole genome enzymatic methylation sequencing (WGMS)

#### Methyl–cytosine conversion and sequencing

Extracted gDNA samples (*n*: Sham=7; PM = 7; Sham-OVA = 7; PM-OVA = 8) underwent QC, enzymatic methyl-cytosine conversion (NEBNext Enzymatic Methyl-seq), and sequencing at the Australian Genome Research Facility (Victoria, Australia). Samples were spiked with non-methylated Lambda DNA to estimate non-conversion, all samples showed methyl-conversion >99%. Methyl-seq libraries were sequenced as 150 bp paired-end reads on Illumina NovaSeq 6000.

#### Data handling

Reads were trimmed with Trim Galore (Babraham Institute), and mapping and methylation calls were carried out using the DRAGEN Methylation Pipeline (v3.10) in a single pass mode against GRCm38 and a spike in Lambda DNA. All samples showed methyl conversion rates >99% and a mean on-target data yield of 111.02 (±SEM = 0.88) Gbps, giving high coverage, as defined by Ziller et al.^106^, of the murine genome (≥42×) per replicate and >290× coverage per treatment group.

#### Sliding window and feature analysis

Data evaluation and statistical analysis were performed using Seqmonk (v1.48.1; Babraham Institute). Data were initially evaluated using 100-CpG sliding window analysis. Percentage DNAm (%DNAm) was calculated per base and binned per window in each replicate, with a minimum threshold of 10 informative CpGs per window e.g., 42 CpG methylation calls per 100-CpG sliding window would give a %DNAm score of 42%. A %DNAm score for a gene was called when a 100 CpG window overlapped an ENSEMBL annotated gene as per the GRCm38 reference genome. DNAm over smaller genomic features (introns, exons, promoter regions and repeat elements) were evaluated using 30-CpG sliding window analyses, with percentage DNA methylation binned per window in each replicate with a minimum threshold of 6 informative CpGs per window. Promoter regions were considered -2000 bp from transcription start sites (TSS) of genes based on the Eponine annotation^107^, intronic and exonic regions were considered those overlapping UCSC intron and exon tracks, and repeat elements were considered those overlapping with RepeatMasker (open-4.0.5). See statistics methods for details on statistical analysis.

### Statistics

#### Biological outcomes

Statistical analysis for lung function tests, immune cell counts and peribronchiolar inflammation was performed using IBM SPSS Statistics Package (version 27.0.1.0) or GraphPad Prism (version 9.5.0 525). All data were tested for normality and appropriate statistical models were applied accordingly. AHR data were analysed using Univariate Analysis of Variance with sex, litter sex skew, litter size and unique maternal ID as covariates. Peribronchiolar inflammation data were analysed using Kruskal–Wallis and Dunn’s post-hoc test. BAL data were analysed with a mixed-effects model with the Geisser-Greenhouse correction and Tukey’s post-hoc test. All data are presented as mean ± standard error of mean unless otherwise stated. All researchers were aware of the group allocation at all stages of the project, with the exception of histological analysis wherein slides were blinded until after images were quantified.

#### Differential gene expression

Data evaluation and statistical analysis were performed using Seqmonk (v1.48.1; Babraham Institute). To define differentially expressed genes (DEGs), DESeq2 was applied to raw counts, with a threshold of *p* < 0.000001 after correction for multiple comparisons with the Benjamini-Hochberg analysis.

#### Differential methylation

Data evaluation and statistical analysis were performed using Seqmonk (v1.48.1; Babraham Institute). To define DMRs, we applied EdgeR across maternal treatment (e.g., Sham v PM, Sham-OVA v PM-OVA) and OVA challenge (Sham v Sham-OVA, PM v PM-OVA) with treatment group and library size as covariates with a threshold of *p* < 0.000001 after correction for multiple comparisons with the Benjamini–Hochberg analysis, and a minimum 10% difference in %DNAm.

### Differentially methylated genes (DMGs)

Statistically determined DMRs were filtered to identify those windows that overlapped ENSEMBL GRCm38 annotated gene sets. The resultant list was deduplicated by ENSEMBL gene code to ensure multiple DMRs across a single gene did not skew the DMG list.

### Key set operations analysis

#### DEGs

We performed set operations analyses across treatment groups for genes shown to be differentially expressed (DEGs) in response to OVA challenge to identify intersections (e.g., ‘Sham v Sham-OVA DEGs’ ∩ ‘PM_2.5_ v PM_2.5_-OVA DEGs’) and differences (e.g., ‘Sham v Sham-OVA DEGs’ - ‘PM_2.5_ v PM_2.5_-OVA DEGs’) across treatment groups.

To perform this, in-house UNIX command line arguments (see Supplementary Methods for exemplar script) cross-comparing ENSEMBL gene codes were used to identify those probes intersecting across and those different between Sham and PM_2.5_ treated cohorts. Resultant DEG sets were referred to as AAD (induced by OVA challenge in both Sham and PM_2.5_ treated offspring), Sham (uniquely induced by OVA in these offspring), and PM_2.5_ (uniquely induced by OVA in these offspring) DEGs, respectively.

#### DMRs

We performed set operations analyses across treatment groups for genes shown to be differentially methylated (DMRs) in response to OVA challenge to identify intersections (e.g., ‘Sham v Sham-OVA DMRs’ ∩ ‘PM_2.5_ v PM_2.5_-OVA DMRs’) and differences (e.g., ‘Sham v Sham-OVA DMRs’ - ‘PM_2.5_ v PM_2.5_-OVA DMRs’) across treatment groups.

To perform this, in-house UNIX command line arguments (see Supplementary Methods for exemplar script) cross-comparing probe locations were used to identify those probes intersecting across and those different between Sham and PM_2.5_ treated cohorts. Resultant DMR sets were referred to as AAD (induced by OVA challenge in both Sham and PM_2.5_ treated offspring), Sham (uniquely induced by OVA in these offspring), and PM_2.5_ (uniquely induced by OVA in these offspring) DMRs, respectively.

#### DEG-DMRs

Source DMR lists were deduplicated by ENSEMBL gene code to provide a list of unique differentially methylated genes (DMGs). Putative interaction between DEGs and DMGs were identified by intersection analysis across identified DEGs and DMGs within AAD, Sham and PM_2.5_ gene sets using in-house UNIX command line arguments (see Supplementary Methods for exemplar script). The resultant DEG-DMG lists infer a putative functional interaction between DEGs and DMGs.

### Gene set enrichment analysis and visualisation

Enrichment analysis was performed on DEGs, DMGs and DEG-DMGs using g:Profiler (version e110_eg57_p18_4b54a898, database updated on 14/09/2023) across Gene Ontology (GO: molecular function) and the Reactome biological pathway with enrichment threshold set at *p* < 0.05. Outputs were visualised with R (v 4.3.1) package ggplot2 (v 3.5.1).

### Cell type gene signature analysis

To elucidate cell-types of interest, we ran our differentially expressed (DEGs), differentially methylated (DMRs), and overlapped differentially expressed and methylated (DEG-DMRs) genes through the Lung Gene Expression Analysis (LGEA) tool^108,109^ against the LungSortedCells database of FACS sorted murine lung cells at PN28. LGEA against LungSortedCells identifies genes selectively expressed from murine lung endothelial, epithelial, immune, mesenchymal and alveolar type-2 cells.

### Reporting summary

Further information on research design is available in the Nature Portfolio Reporting Summary linked to this article.

## Supplementary information

Supplementary material

Reporting Summary

## Data Availability

Data are publicly available in a repository. Numerical source data for graphs and charts are uploaded to Figshare and are available at 10.6084/m9.figshare.28386518.v1^110^. The RNA-Seq & WGMS raw and processed data files generated during and analysed during the current study are available in the NCBI Gene Expression Omnibus (GEO) repository with the following accession codes: RNA-Seq: GSE285128. WGMS: GSE285243.
