# Supplementary material for: Developmental air pollution exposure augments airway hyperreactivity, alters transcriptome, and DNA methylation in female adult progeny
Source: Commun Biol. 2025 Mar 8;8:400. doi: 10.1038/s42003-025-07835-0 (PMC11890619; doi:10.1038/s42003-025-07835-0)
Supplement: Supplementary file 1 — Supplementary material [file 42003_2025_7835_MOESM1_ESM.pdf]

Supplementary methods:

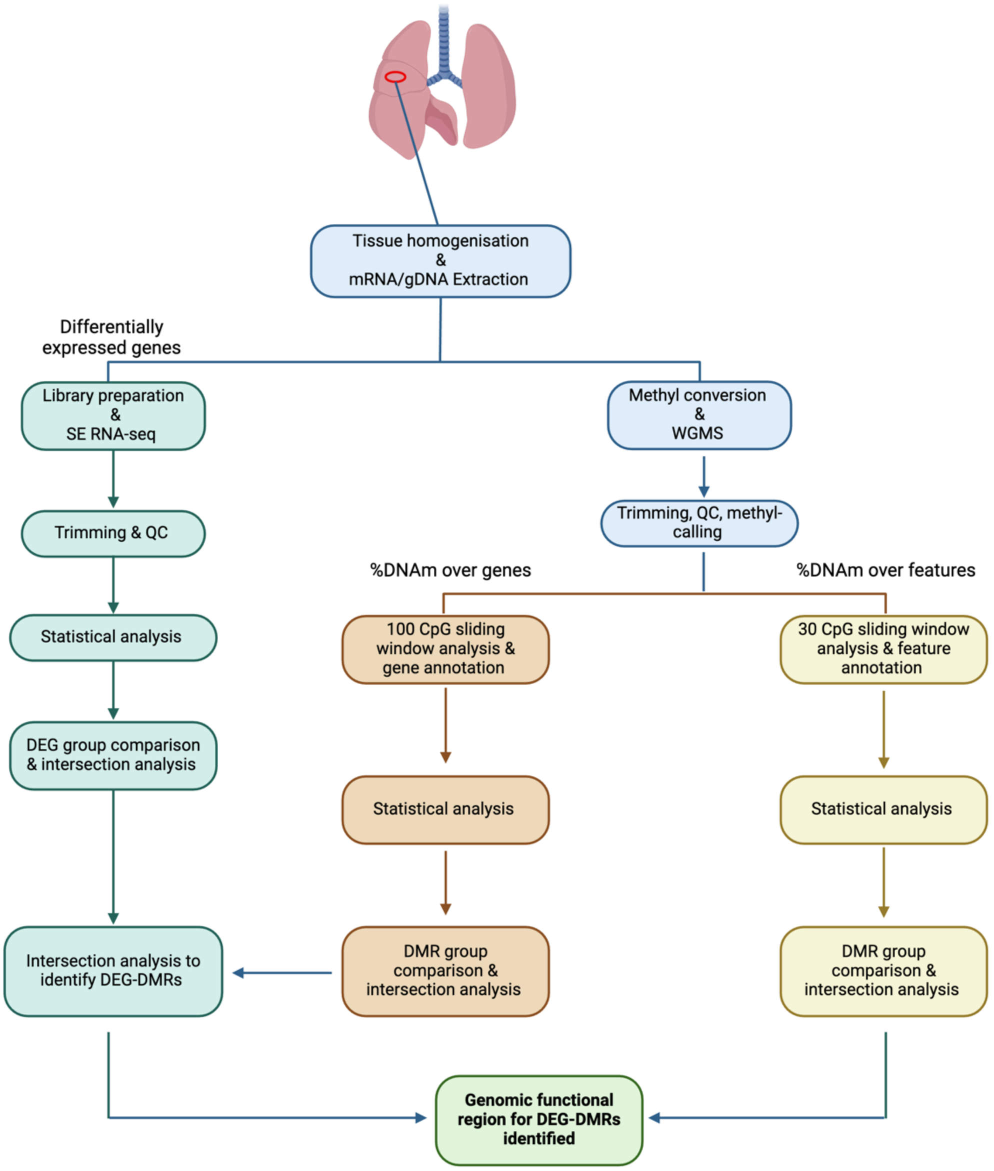

Bioinformatic pipeline.

Exemplar gene filtering code:

```
$file1="DESeq_1.txt"
$file2="DESeq_6.txt"
$unique_file1="UV_1.txt"
$unique_file2="UV_2.txt"
$duplicate_file1="DF_1.txt"
$duplicate_file2="DF_2.txt"
$cut -f 3 $file1 > ${file1}.col3
```

```
$cut -f 3 $file2 > ${file2}.col3
$sort ${file1}.col3 ${file2}.col3 | uniq -d > ${duplicate_file1}.col3
$sort ${file1}.col3 ${file2}.col3 | uniq -u > ${unique_file1}.col3
$awk 'FNR==NR{a[$1]; next} $3 in a' ${duplicate_file1}.col3 $file1 > ${duplicate_file1}
$awk 'FNR==NR{a[$1]; next} $3 in a' ${duplicate_file2}.col3 $file2 > ${duplicate_file2}
$awk 'FNR==NR{a[$1]; next} $3 in a' ${unique_file1}.col3 $file1 > ${unique_file1}
$awk 'FNR==NR{a[$1]; next} $3 in a' ${unique_file2}.col3 $file2 > ${unique_file2}
$unique_count_file1=$(wc -l < $unique_file1)
$unique_count_file2=$(wc -l < $unique_file2)
$duplicate_count_file1=$(wc -l < $duplicate_file1)
$duplicate_count_file2=$(wc -l < $duplicate_file2)
$rm *.col3
```

Cellular deconvolution of transcriptomic signal

We developed a signature matrix comprising 16 cell types: ATI epithelial cells, ATII epithelial cells, alveolar macrophages, B cells, capillary endothelial cells, CD4 T cells, CD8 T cells, dendritic cells, fibroblasts, granulocytes, interstitial macrophages, monocytes, multiciliated/deuterosomal cells, NK cells, secretory endothelial cells, and venous endothelial cells. This matrix was created from healthy subsets of a single-cell mouse atlas (n=59). Initially, raw counts of 200 randomly selected cells from each cell type were extracted to generate the reference matrix. We then uploaded this reference matrix to CIBERSORTx (Newman et al., 2015; Newman et al., 2019), where we generated a custom single-cell RNA sequencing (scRNA-seq) signature matrix. We disabled quantile normalization and maintained all other parameters at their default settings. Subsequently, the acquired signature matrix was used to predict the cell type composition of a bulk RNA-seq dataset within CIBERSORTx. The bulk RNA-seq data underwent count per million (CPM) normalization according to CIBERSORTx guidelines. We imputed cell fractions with batch corrections enabled and permutations set to 100 for significance analysis. Results are expressed as proportions of the whole.

*M., & Alizadeh, A. A. (2015). Robust enumeration of cell subsets from tissue expression profiles. Nature Methods, 12(5), 453-457. https://doi.org/10.1038/nmeth.3337

*Newman, A. M., Steen, C. B., Liu, C. L., Gentles, A. J., Chaudhuri, A. A., Scherer, F., Khodadoust, M. S., Esfahani, M. S., Luca, B. A., Steiner, D., Diehn, M., & Alizadeh, A. A. (2019). Determining cell type abundance and expression from bulk tissues with digital cytometry. Nature Biotechnology, 37(7), 773-782. https://doi.org/10.1038/s41587-019-0114-2

Supplementary Figures:

A

Component Quantity	µg/m³
Chloride	0.44 ± 0.03
Nitrite	0.2 ± 0.01
Nitrate	3.11 ± 0.25
Sulphate	8.29 ± 0.48
Sodium	2.62 ± 0.14
Ammonium	1.71 ± 0.13
Potassium	0.24 ± 0.02
Organic carbon	7.86 ± 0.51
Elemental carbon	4.46 ± 0.20

B

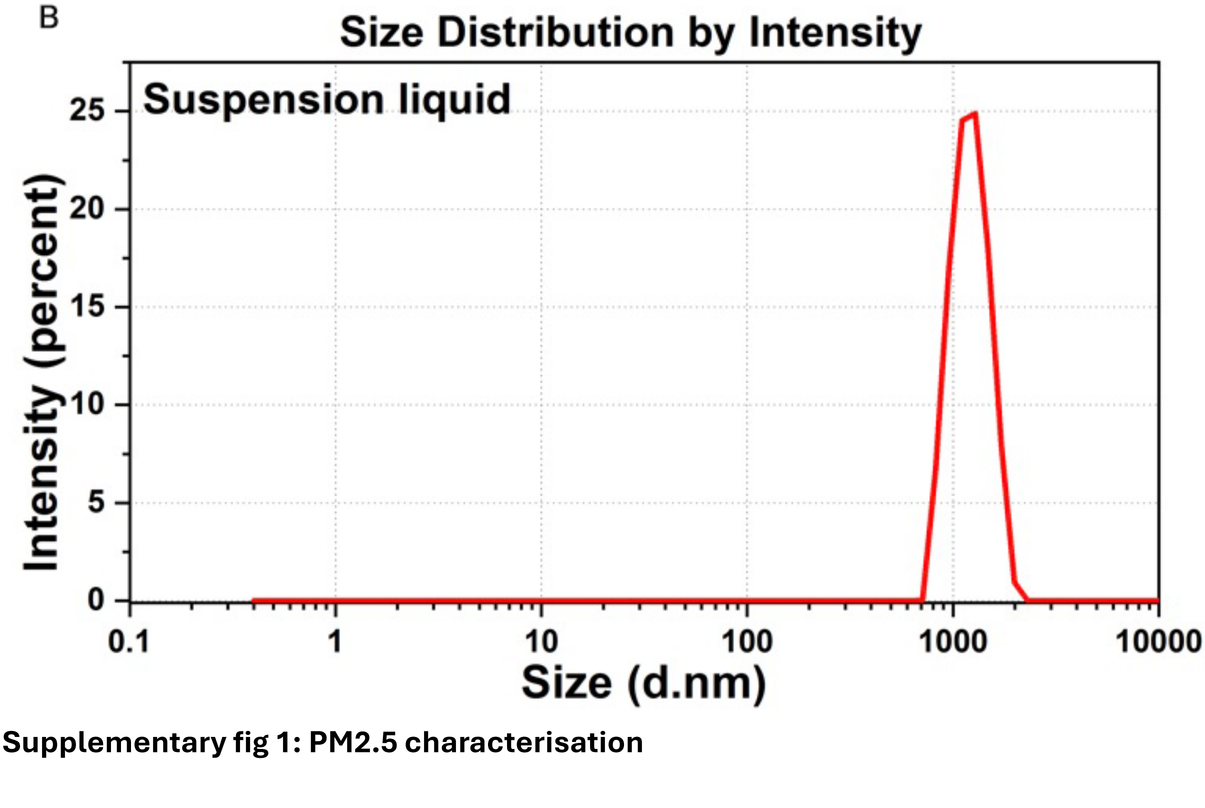

Supplementary fig 1: PM2.5 characterisation

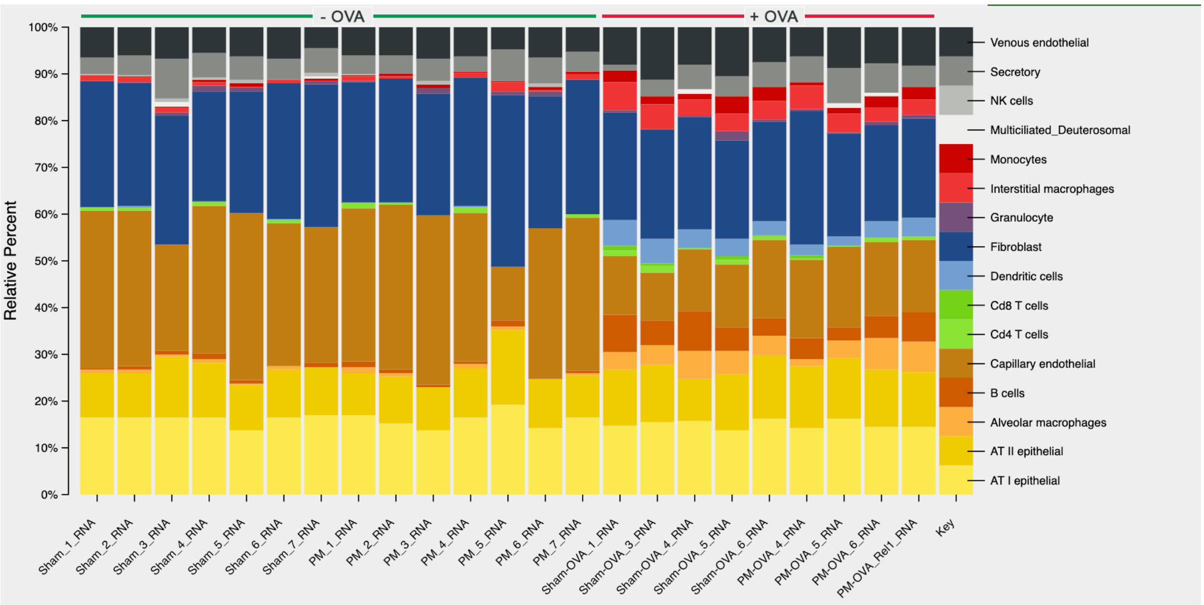

Supp figure 2: Cellular deconvolution of transcriptomic data. Each column represents a single replicate. Green line above column indicates non-AAD induced (-OVA), whilst red line above column indicates AAD induced (+OVA). It is evident that OVA stimulation induces a dendritic cell mediated Type II immune response with no diff between Sham and PM effect.

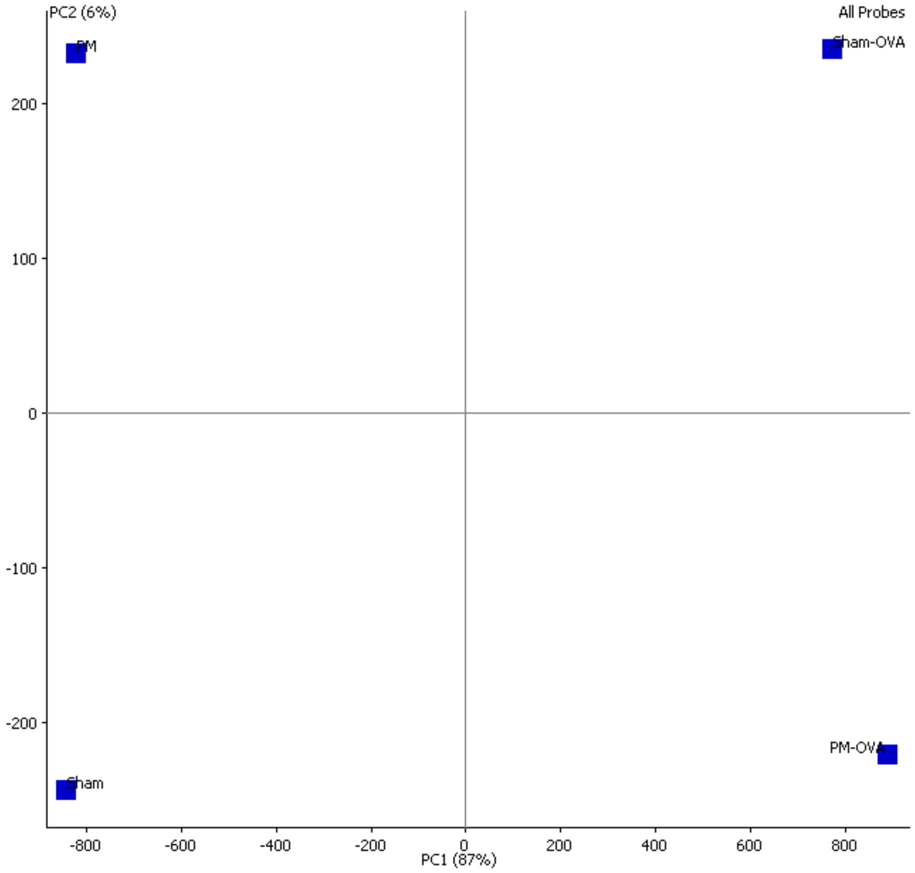

Supplementary figure 3: principal component analysis of whole genome methylation grouped by treatment (n=7-8 per group)

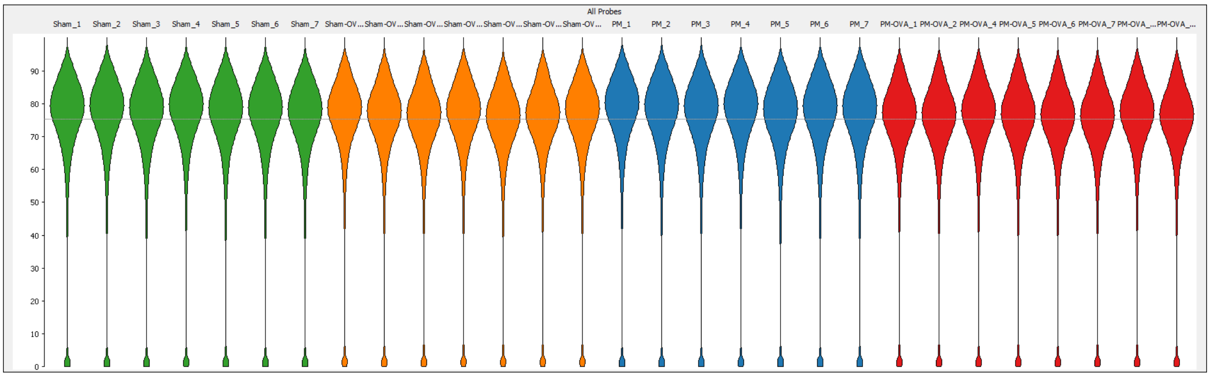

Supplementary figure 4: whole genome methylation distribution of each replicate (green=sham, orange=sham-ova, blue=pm, red=pm-ova)

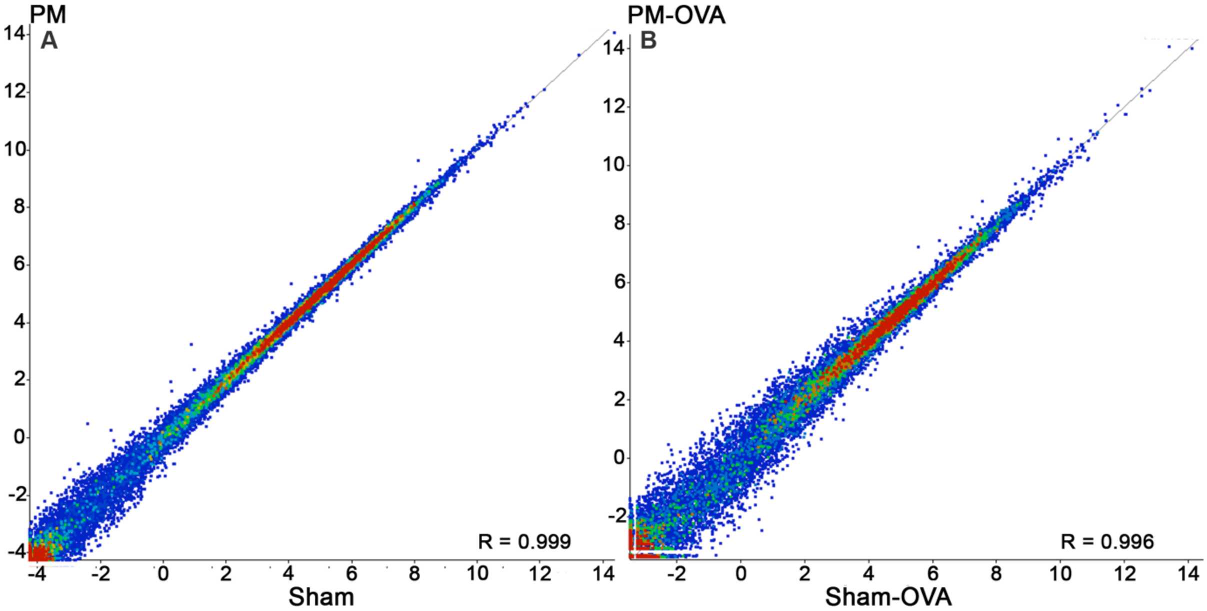

Supplementary figure 5: Scatter plot showing %DNAm scores across 100 CpG sliding windows when comparing (a) Sham v PM; and (b) Sham-OVA v PM-OVA

Supp Table 1.1

Ovalbumin Induced Transcriptome Changes Unique to Sham					
Upregulated			Downregulated		
GO.ID	Description	p.Val	GO.ID	Description	p.Val
GO:0005515	protein binding	4.96E-20	GO:0005515	protein binding	2.42E-66
GO:0003824	catalytic activity	1.75E-15	GO:0005488	binding	1.33E-58
GO:0005488	binding	1.31E-13	GO:0043167	ion binding	6.32E-32
GO:0019899	enzyme binding	1.53E-08	GO:0043169	cation binding	3.15E-21
GO:0044877	protein-containing complex binding	5.09E-07	GO:0046872	metal ion binding	3.4E-20
GO:0042802	identical protein binding	9.66E-07	GO:0019899	enzyme binding	3.31E-15
GO:0140640	catalytic activity, acting on a nucleic acid	1.0831E-06	GO:0140096	catalytic activity, acting on a protein	7.7E-12
GO:0030554	adenyl nucleotide binding	4.4695E-06	GO:0008092	cytoskeletal protein binding	1.26E-09
GO:0032559	adenyl ribonucleotide binding	5.6662E-06	GO:0140110	transcription regulator activity	1.37E-09
GO:0016787	hydrolase activity	1.2499E-05	GO:0019904	protein domain specific binding	4.12E-09
GO:0017076	purine nucleotide binding	1.5103E-05	GO:0008134	transcription factor binding	7.86E-09
GO:0005524	ATP binding	1.7214E-05	GO:0017076	purine nucleotide binding	9.91E-09
GO:0032553	ribonucleotide binding	1.921E-05	GO:0043168	anion binding	2.41E-08
GO:0032555	purine ribonucleotide binding	2.0703E-05	GO:0003824	catalytic activity	4.8E-08
GO:0035639	purine ribonucleoside triphosphate binding	4.5805E-05	GO:0035639	purine ribonucleoside triphosphate binding	5.09E-08
GO:0140657	ATP-dependent activity	5.47E-05	GO:0032555	purine ribonucleotide binding	5.38E-08
GO:0097367	carbohydrate derivative binding	0.00012431	GO:0097367	carbohydrate derivative binding	7.67E-08
GO:0000166	nucleotide binding	0.0001731	GO:0000166	nucleotide binding	9.24E-08
GO:1901265	nucleoside phosphate binding	0.0001731	GO:1901265	nucleoside phosphate binding	9.24E-08
GO:0031625	ubiquitin protein ligase binding	0.00029249	GO:0032553	ribonucleotide binding	1.21E-07
GO:0043168	anion binding	0.00032895	GO:0044877	protein-containing complex binding	4.65E-07
GO:0044389	ubiquitin-like protein ligase binding	0.00100501	GO:0030554	adenyl nucleotide binding	6.89E-07
GO:0003697	single-stranded DNA binding	0.0010577	GO:0032559	adenyl ribonucleotide binding	3.72E-06
GO:0016887	ATP hydrolysis activity	0.00207431	GO:0005524	ATP binding	5.36E-06
GO:0140098	catalytic activity, acting on RNA	0.00426691	GO:0036094	small molecule binding	5.9E-06
GO:0140097	catalytic activity, acting on DNA	0.00446196	GO:0016740	transferase activity	7.68E-06
GO:0003682	chromatin binding	0.00462577	GO:0003779	actin binding	1.23E-05
GO:0032135	DNA insertion or deletion binding	0.00545133	GO:0019901	protein kinase binding	3.65E-05
GO:0016462	pyrophosphatase activity	0.00774552	GO:0061629	RNA polymerase II-specific DNA-binding transcription fa	5.09E-05
GO:0043167	ion binding	0.00799649	GO:0140297	DNA-binding transcription factor binding	5.95E-05
GO:0016817	hydrolase activity, acting on acid anhydrides	0.00803601	GO:0046332	SMAD binding	6.47E-05
GO:0016818	hydrolase activity, acting on acid anhydrides, in phosph	0.00803601	GO:0019900	kinase binding	9.22E-05
GO:0017111	ribonucleoside triphosphate phosphatase activity	0.00813963	GO:0003712	transcription coregulator activity	0.000167
GO:0004812	aminoacyl-tRNA ligase activity	0.01040207	GO:0003677	DNA binding	0.00018
GO:0016875	ligase activity, forming carbon-oxygen bonds	0.01040207	GO:0000987	cis-regulatory region sequence-specific DNA binding	0.000197
GO:0098772	molecular function regulator activity	0.01519464	GO:0016301	kinase activity	0.000246
GO:0008094	ATP-dependent activity, acting on DNA	0.01790676	GO:0016772	transferase activity, transferring phosphorus-containing	0.000319
GO:0036094	small molecule binding	0.0191269	GO:0019787	ubiquitin-like protein transferase activity	0.00037
GO:0140299	small molecule sensor activity	0.02333926	GO:0003700	DNA-binding transcription factor activity	0.00049
GO:0032142	single guanine insertion binding	0.03101174	GO:0000978	RNA polymerase II cis-regulatory region sequence-specif	0.000696
GO:0032139	dinucleotide insertion or deletion binding	0.03101174	GO:0001067	transcription regulatory region nucleic acid binding	0.000699
			GO:0043565	sequence-specific DNA binding	0.000705
			GO:0004842	ubiquitin-protein transferase activity	0.000713
			GO:0003713	transcription coactivator activity	0.000734
			GO:0016773	phosphotransferase activity, alcohol group as acceptor	0.000783
			GO:0004675	transmembrane receptor protein serine/threonine kina	0.000897
			GO:0000976	transcription cis-regulatory region binding	0.000962
			GO:0016755	aminoacyltransferase activity	0.00111
			GO:0097159	organic cyclic compound binding	0.001305
			GO:1990837	sequence-specific double-stranded DNA binding	0.001556
			GO:0003690	double-stranded DNA binding	0.001686
			GO:0042802	identical protein binding	0.001763
			GO:0005024	transforming growth factor beta receptor activity	0.002102
			GO:0000981	DNA-binding transcription factor activity, RNA polymer	0.002161
			GO:0019955	cytokine binding	0.002731
			GO:0001227	DNA-binding transcription repressor activity, RNA polyr	0.002851
			GO:1901363	heterocyclic compound binding	0.003332
			GO:0060589	nucleoside-triphosphatase regulator activity	0.003377
			GO:0060090	molecular adaptor activity	0.003471
			GO:0001217	DNA-binding transcription repressor activity	0.004164
			GO:0000977	RNA polymerase II transcription regulatory region seque	0.005014
			GO:0016922	nuclear receptor binding	0.005022
			GO:0030695	GTPase regulator activity	0.005531
			GO:0005543	phospholipid binding	0.006764
			GO:0004672	protein kinase activity	0.008108
			GO:0019199	transmembrane receptor protein kinase activity	0.009189
			GO:0061659	ubiquitin-like protein ligase activity	0.010613
			GO:0015631	tubulin binding	0.013741
			GO:0004674	protein serine/threonine kinase activity	0.016407
			GO:0061630	ubiquitin protein ligase activity	0.017219
			GO:0048185	activin binding	0.019708
			GO:0017002	activin receptor activity	0.023144
			GO:0008289	lipid binding	0.023222
			GO:0044389	ubiquitin-like protein ligase binding	0.023233
			GO:0030674	protein-macromolecule adaptor activity	0.027836
			GO:0016791	phosphatase activity	0.0294
			GO:0031625	ubiquitin protein ligase binding	0.030831
			GO:0004721	phosphoprotein phosphatase activity	0.031291
			GO:0005025	transforming growth factor beta receptor activity, type	0.037745
			GO:0016361	activin receptor activity, type I	0.037745
			GO:0001025	RNA polymerase III general transcription initiation facto	0.037745
			GO:0005160	transforming growth factor beta receptor binding	0.045946
			GO:0051219	phosphoprotein binding	0.045968
			GO:0030234	enzyme regulator activity	0.04932

Supp Table 1.2

Ovalbumin Induced Transcriptome Changes Unique to PM					
Upregulated			Downregulated		
GO.ID	Description	p.Val	GO.ID	Description	p.Val
	NO RESULT		GO:0005488	binding	1.77E-05
			GO:0043167	ion binding	6.97E-05
			GO:0005515	protein binding	0.000243
			GO:0016301	kinase activity	0.007143
			GO:0016772	transferase activity, transferring phosphorus-containing groups	0.015199
			GO:0043169	cation binding	0.023067
			GO:0003824	catalytic activity	0.031956
			GO:0046872	metal ion binding	0.033421
			GO:0097367	carbohydrate derivative binding	0.045923
			GO:0016773	phosphotransferase activity, alcohol group as acceptor	0.049819

Supp Table 1.3

Ovalbumin Induced Methylome Changes Unique to Sham

Hypermethylated			Hypomethylated		
GO:0005515	protein binding	5.07E-37	GO:0005515	protein binding	4.59E-06
GO:0005488	binding	3.85E-27	GO:0005488	binding	4.09E-05
GO:0044877	protein-containing complex binding	1.44E-12	GO:0005096	GTPase activator activity	8.69E-05
GO:0019904	protein domain specific binding	2.48E-10	GO:0030695	GTPase regulator activity	9.86E-05
GO:0030695	GTPase regulator activity	4.98E-10	GO:0060589	nucleoside-triphosphatase regulator activity	0.000109
GO:0060589	nucleoside-triphosphatase regulator activity	6.34E-10	GO:0005085	guanyl-nucleotide exchange factor activity	0.00068
GO:0019899	enzyme binding	7.45E-10	GO:0008047	enzyme activator activity	0.011021
GO:0019900	kinase binding	2.11E-09			
GO:0019901	protein kinase binding	1.26E-08			
GO:0098772	molecular function regulator activity	5.77E-08			
GO:0004672	protein kinase activity	2.47E-07			
GO:0030234	enzyme regulator activity	7.49E-07			
GO:0043167	ion binding	8.64E-07			
GO:0016773	phosphotransferase activity, alcohol group as acceptor	1.0764E-06			
GO:0042802	identical protein binding	3.2334E-06			
GO:0004674	protein serine/threonine kinase activity	4.3289E-06			
GO:0016301	kinase activity	4.6357E-06			
GO:0043168	anion binding	1.167E-05			
GO:0005096	GTPase activator activity	1.6635E-05			
GO:0035591	signaling adaptor activity	2.3172E-05			
GO:0005085	guanyl-nucleotide exchange factor activity	5.5128E-05			
GO:0016772	transferase activity, transferring phosphorus-containinį	7.2798E-05			
GO:0005102	signaling receptor binding	9.5125E-05			
GO:0140096	catalytic activity, acting on a protein	0.00010877			
GO:0140110	transcription regulator activity	0.00014927			
GO:0036094	small molecule binding	0.00028251			
GO:0097367	carbohydrate derivative binding	0.00045396			
GO:0051015	actin filament binding	0.00066301			
GO:0008092	cytoskeletal protein binding	0.00092921			
GO:0008047	enzyme activator activity	0.00106509			
GO:0032555	purine ribonucleotide binding	0.00139482			
GO:0016740	transferase activity	0.00145766			
GO:0003779	actin binding	0.00159082			
GO:0032553	ribonucleotide binding	0.00188354			
GO:0008289	lipid binding	0.00197809			
GO:0017076	purine nucleotide binding	0.00203736			
GO:1990782	protein tyrosine kinase binding	0.00212602			
GO:0046872	metal ion binding	0.00255922			
GO:0060090	molecular adaptor activity	0.00314696			
GO:0046875	ephrin receptor binding	0.00325207			
GO:0032559	adenyl ribonucleotide binding	0.00355289			
GO:0001228	DNA-binding transcription activator activity, RNA polymerase II-specific	0.00363088			
GO:0043169	cation binding	0.00410483			
GO:0001216	DNA-binding transcription activator activity	0.00438928			
GO:1990837	sequence-specific double-stranded DNA binding	0.00468217			
GO:0030554	adenyl nucleotide binding	0.00541309			
GO:0001221	transcription coregulator binding	0.00688287			
GO:0035639	purine ribonucleoside triphosphate binding	0.0075575			
GO:0030229	very-low-density lipoprotein particle receptor activity	0.00780791			
GO:0003713	transcription coactivator activity	0.00897869			
GO:1901265	nucleoside phosphate binding	0.01004552			
GO:0000166	nucleotide binding	0.01004552			
GO:0005524	ATP binding	0.01108154			
GO:0003690	double-stranded DNA binding	0.01126477			
GO:0000976	transcription cis-regulatory region binding	0.01247418			
GO:0019903	protein phosphatase binding	0.01267079			
GO:0001067	transcription regulatory region nucleic acid binding	0.01360663			
GO:0043565	sequence-specific DNA binding	0.01460249			
GO:0030159	signaling receptor complex adaptor activity	0.01584935			
GO:0050431	transforming growth factor beta binding	0.01606281			
GO:0044325	transmembrane transporter binding	0.02544974			
GO:0042803	protein homodimerization activity	0.02574964			
GO:0045309	protein phosphorylated amino acid binding	0.02734937			
GO:0003712	transcription coregulator activity	0.03492942			
GO:0030674	protein-macromolecule adaptor activity	0.03515297			
GO:0035091	phosphatidylinositol binding	0.03557789			
GO:0019902	phosphatase binding	0.03621997			
GO:0030228	lipoprotein particle receptor activity	0.03681312			
GO:0106310	protein serine kinase activity	0.04521251			
GO:0003700	DNA-binding transcription factor activity	0.04774448			
GO:0000977	RNA polymerase II transcription regulatory region sequence-specific DNA binding	0.04811508			

Supp Table 1.4

Ovalbumin Induced Methylome Changes Unique to PM					
Hypermethylated			Hypomethylated		
GO:0005488	binding	0.00097997	GO:0005515	protein binding	2.68E-08
GO:0001223	transcription coactivator binding	0.00674807	GO:0005488	binding	7.42E-07
GO:0005515	protein binding	0.01079884	GO:0019899	enzyme binding	1.76E-05
			GO:0043565	sequence-specific DNA binding	0.005268
			GO:0003677	DNA binding	0.0058
			GO:0043167	ion binding	0.009176
			GO:0004726	non-membrane spanning protein tyrosine phosphatase activity	0.016038
			GO:0000987	cis-regulatory region sequence-specific DNA binding	0.035859
			GO:0003690	double-stranded DNA binding	0.038199
			GO:1990837	sequence-specific double-stranded DNA binding	0.039868
			GO:0140110	transcription regulator activity	0.045212
			GO:0000976	transcription cis-regulatory region binding	0.049286

Supp tables 2

Shared AAD - RNA

Cell type	SEG	P-value	Graphical annotation
ENDO	*2900026A02RIK; 4930503L19RIK; ACKR3; ADGRF5; ADGRL1; ADGRL4; AGRN; ANKRD50; ARAP2; ARHGEF12; ARMCX1; ARMCX4; BICD2; BMPR2; BST2; BTBD3; CALCRL; CCM2L; CD93; CDAN1; CLIC5; CRACR2B; CYTH3; CYYR1; DDAH2; DLC1; EFNB2; EHD2; ENG; ENPP4; FBLN2; FGD5; FZD4; GBP7; GIMAP8; GRRP1; HPGD; ITGA1; ITGA6; JAM2; KANK3; KITL; NAV1; NHLRC2; PCNX; PKN3; PLEKHO1; PPM1F; PTPRB; PXDN; QK; RAMP2; RHOC; S100A16; S1PR1; SCN7A; SLC39A6; SNN; ST3GAL2; THBD; TJP1; TMOD2; TNFSF10; TRIM16; ZFP641*	0.0004	***
EPI	*1110065P20RIK; ACAT2; ACSS1; AKAP5; ALCAM; ALDH1A7; ANPEP; CAT; CCZ1; CDC42BPG; CGN; CHIA1; CHIL1; CHKA; CKMT1; CPM; DDT; EMC6; EPCAM; EXPH5; FDX1; FZD5; GALK1; GCA; GLRX; HDC; HINT2; IFT20; IGIP; KCNE2; KLHDC7A; LMO7; LYPD2; LYZ1; ME1; MLPH; MT1; NDNF; NDUFC2; NKX2-1; NPC2; NUPR1; PDZK1IP1; PIGR; PIGYL; POLR1C; PON1; PON3; PPP1R26; PPP1R9A; RAB11FIP1; RAI14; SCGB3A1; SEC14L3; SEC14L4; SELENBP1; SFTPA1; SFTPD; SGPP2; SLC26A9; SLC6A14; SMIM22; SOD2; SPINT2; STARD10; TFF2; TMEM107; TST; VAMP8; ZFP612*	0.0000958	****
IMMUNE	*2410006H16RIK; ABRACL; AIF1; ARHGDIB; ARL5C; ARRB2; CCL22; CENPA; CNP; DCAF15; GPSM3; HCLS1; LGALS1; LTB; MAP4K1; ME2; NFKBIE; NHP2; PAG1; PFN1; PIK3R5; PSMB8; PTPRC; PYCARD; RAC2; RBM38; RPA2; RPLP0; SELPLG; TALDO1; UBA52;*	0.0009	***
MES	*ACVR2A; ADRB3; ARHGAP21; ARHGEF17; ATF5; BACE1; BMP5; BMPR1A; CCL11; CFB; CHST3; CMKLR1; COL14A1; COL6A2; COPZ2; CPXM1; CTSF; CTSL; CYGB; EFEMP2; FAM49A; FBXO30; FMO2; FXYD1; FZD1; HHIP; IGFBP3; ITGA8; KDELR3; LEPR; LIMCH1; LRP4; MACF1; MDK; MS4A4D; NEDD4; NID1; PAPSS2; PDGFRA; PDIA4; PKD2; PLXDC2; PMEPA1; PREX2; RAD50; RBP1; RECK; SCN3A; SCUBE2; STARD13; SYDE2; TACC1; TCAF1; TFPI; TGFBR3; TNFRSF19; TNS1; TWSG1; UGDH; VCL; VLDLR; ZBTB10; ZYX;*	0.0003	***

Sham AAD - RNA

Cell type	SEG	P-value	Graphical annotation
ENDO	*1500015A07RIK; ABI3; ACER2; ACKR2; ACVRL1; ADGRL2; ADRB1; ADRB2; AMIGO2; APLNR; APOLD1; AQP1; CAR4; CASZ1; CCRL2; CLEC14A; CLEC2D; DENND3; EDN1; EDNRB; EGFL7; EHD4; EMCN; EPHB4; ETS2; FIGNL2; FMNL3; FSCN1; GIMAP6; GRAP; HPGD; ID3; ITGA5; JUN; JUP; KDR; KIT; KLF2; KLF4; KLHL5; LIMS2; LY6A; LYVE1; MID2; MYZAP; NIPAL3; NOTCH4; NPTX1; OLFML2A; PALMD; PHF11D; PLAU; PLSCR3; RASGEF1A; RASSF1; RNASE1; RNF144A; RTP3; SCN3B; SEMA3C; SEMA3G; SEMA6A; SERPINE1; SGK1; SHE; SMAD7; SORBS3; SOX17; SOX7; SSTR4; STARD8; TAL1; TCN2; TEK; TGFB1I1; TMEM100; TRIB2; TSPAN18; TUBB2A*	1.98E-07	****
EPI	*1110032A03RIK; 2610028H24RIK; AA986860; ABCD3; ADI1; AGER; ARHGDIG; ATP11A; BRI3; CCKAR; CDS1; CEP170B; CES1D; CISD3; CLDN18; CLDN23; CNDP2; CXADR; D230025D16RIK; DSTN; ESRP2; ETV5; FAT1; FGFBP1; FOXA1; GPRC5A; GRB7; HC; HMGN1; HP; IAH1; ID2; IRX5; ITIH4; LAPTM4B; LRRC8E; LYZ2; MAL; MARVELD3; MFAP3L; MID1IP1; MLYCD; MPC1; MUC1; PAFAH2; PFKFB2; PPP1R14C; PTGR1; PTPRF; REEP6; RENBP; RIPK4; RMDN2; SCNN1A; SDC1; SFTA2; SFTPC; SIVA1; SLC31A1; SLC4A5; SLCO4C1; SOWAHC; SPRR1A; SPRY2; TBC1D30; TC2N; TCTEX1D4; TFCP2L1; TFRC; TMEM243; VCPKMT*	5.91E-05	****
IMMUNE	*ACTR3; AI662270; ALYREF; ARHGAP4; ARHGAP9; BTG1; CD2; CDKN2D; DENND4B; ERI1; FMNL1; GPR65; HCST; NCKAP1L; OSM; PABPC1; PF4; PSD4; RGS1; RINL; RPSA; SAP30; SMAP2; VAMP1; ZFP36L2*	0.0257	*
MES	*2700081O15RIK; ABCA8A; ADRB3; AGO1; AKAP12; ATF5; AXIN2; BGN; BMP3; C1QTNF6; C7; CCDC80; CDC42EP4; CFH; CREB3L1; DKK3; DNAJB4; DSEL; DUSP1; EVI5; FBLIM1; FBLN1; FHL1; FKBP14; FZD7; GPX7; GSTM2; H6PD; HEYL; KDELR3; LTBP4; LUM; MAGED1; MAMDC2; MAP1A; MERTK; MFAP5; MFGE8; MGP; MMP11; MMP14; MORF4L2; MR1; MYLK; NEDD4; NEXN; NPNT; OLFML1; OSMR; P3H3; PDP1; PEG3; PIK3R1; PODN; PPP1R3C; RAP2A; RCN3; SERPINE2; SPARC; SPON1; SVEP1; TBX4; TGFBR3; TNS3; UGDH; VCL; VIPR2; ZFP354C*	1.99E-05	****

PM AAD - RNA

Cell type	SEG	P-value	Graphical annotation
ENDO	*ARHGAP27; FAM124B; LUZP1; SNRK;*	0.7853	ns
EPI	*ARHGDIG; ATP8A1; BPIFB1; CFAP126; CRB3; CTXN1; FOXJ1; GSTT2; GSTZ1; IL18R1; LRRC23; PPP1R9A; SCD2; SNHG11; SNX25; TCTEX1D4; TSPAN1; VPREB3; WFDC2;*	6.28E-06	****
IMMUNE	*NOC2L; PARVG; SNRNP25; UCP2;*	0.1645	ns
MES	*ATP2B4; CALD1; ENTPD2; FITM2; LAMB1; MMP11; MSLN; PODN; SDC2; SERINC5*	0.0346	*

Shared AAD - WGMS

Cell type	SEG	P-value	Graphical annotation
ENDO	*ADCY4 ADGRE5 AMIGO2 ARHGAP27 ARHGEF12 ARL4D BCL6B BTBD3 CASKIN2 CASZ1 CLIC5 DLC1 DLL4 DOCK6 ECE1 EFNA1 EGFL7 EHD4 ELK3 ENG EPAS1 ETS2 FLT1 GATA2 GIT2 GPR182 HLX HYAL2 ITGA1 KIT KLF7 KLHL5 MAP7D1 NAV1 NOSTRIN NOTCH1 PECAM1 PLK2 PLXND1 PRICKLE1 QK RASGRP3 RHOC SEMA3F SEMA7A SGK1 SH2D3C SHANK3 SIPA1 SLC9A3R2 SMAD7 SOX7 SRGN TMEM100 TMEM88 TSPAN13 USHBP1*	1.49E-24	****
EPI	*SEC14L4 SFN TRAF1*	0.9977	ns
IMMUNE	*AI467606 ARHGAP30 ARHGAP9 BIN2 CD2 CD52 CORO1A DOK2 FAM107B FERMT3 FMNL1 FYB IKZF3 MOB3A MYC OSM PIK3CG PIP4K2A PTPRC RASAL3 RPA2 SASH3 SELL SPN STK17B TNFAIP3*	3.29E-13	****
MES	*2700081O15RIK ANTXR1 CBLB DUSP1 FZD7 GSTM2 LIX1L PCOLCE RAD50 SERPINF1 TBC1D2B TNS1*	0.2315	ns

Sham AAD - WGMS

Cell type	SEG	P-value	Graphical annotation
ENDO	*ACVRL1; AHR; ANKRD50; AQP1; ARHGAP23; ARHGEF12; ARHGEF15; BMPR2; CASZ1; CCM2L; CDH5; CLIC5; CNN3; COL4A1; CTTNBP2NL; CYTH3; CYYR1; DENND3; DLC1; ECE1; EFNA1; EFNB2; EGFL7; EHD4; ENG; EPAS1; EPHB4; ETS2; FLNB; FMNL3; GATA2; GNG11; HILPDA; KITL; KLF7; LYVE1; MYZAP; NAV1; NHLRC2; PALMD; PCDH1; PCDH12; PLXNA1; PLXND1; PODXL; PRX; QK; RAPGEF3; RASIP1; RGL1; SEMA3F; SEMA6A; SGK1; SH2B3; SLC9A3R2; ST3GAL2; TBXA2R; TJP1; TMEM184B*	2.65E-29	****
EPI	*AI661453; ETV5; KRT8; PAFAH2; SCD2; SLC43A2; TPM1*	0.7849	ns
IMMUNE	*ARHGAP9; CYFIP2; FAM107B; FMNL1; FYB; LTB; PSD4; PSMB8; RAC2; SPATA13; UCP2*	0.0008	***
MES	*2700081O15RIK; ARHGEF17; BMP5; CSRP1; FHOD1; HSPB8; LAMC1; LRP1; MACF1; MMP14; MMP2; MYLK; NEDD4; NPNT; NREP; OLFML3; PBX1; PI16; PIK3R1; RCN3; SNX33; TGFB3; TGFBR3; TNS1; TNS3; TUBB4A*	4.81E-07	****

PM AAD - WGMS

Cell type	SEG	P-value	Graphical annotation
ENDO	*CASZ1 CYTH3 EPAS1 FIGNL2 MAP7D1 MYO10 NAV1 PODXL QK SH2B3 SIPA1 THSD1*	0.0006	***
EPI	*NPC2 RAB11FIP1 SOCS2*	0.7635	ns
IMMUNE	*CD37 CNP FAM107B IKZF3*	0.0675	ns
MES	*HHIP LRP1 MXRA8 PMEPA1 SCARF2 TBC1D2B*	0.1603	ns

Shared AAD – DEG-DMRs

Cell type	SEG	P-value	Graphical annotation
ENDO	*ARHGEF12; BTBD3; CLIC5; DLC1; ENG; ITGA1; NAV1; QK; RHOC;*	0.0004	***
EPI	*SEC14L4;*	0.9001	ns
IMMUNE	*PTPRC; RPA2;*	0.2148	ns
MES	*RAD50; TNS1;*	0.6167	ns

Sham AAD – DEG-DMRs

Cell type	SEG	P-value	Graphical annotation
ENDO	*ACVRL1 AQP1 CASZ1 DENND3 EGFL7 EHD4 EPHB4 ETS2 FMNL3 LYVE1 MYZAP PALMD SEMA6A SGK1*	6.95E-09	****
EPI	*ETV5 PAFAH2*	0.5744	ns
IMMUNE	*ARHGAP9 FMNL1 PSD4*	0.0373	*
MES	*2700081O15RIK MMP14 MYLK NEDD4 NPNT PIK3R1 RCN3 TGFBR3 TNS3*	7.48E-05	****

PM AAD – DEG-DMRs

Cell type	SEG	P-value	Graphical annotation
ENDO	*NO HITS*		
EPI	*NO HITS*		
IMMUNE	*NO HITS*		
MES	*NO HITS*		
