# Supplementary material for: Developmental air pollution exposure augments airway hyperreactivity, alters transcriptome, and DNA methylation in female adult progeny
Source: Commun Biol. 2025 Mar 8;8:400. doi: 10.1038/s42003-025-07835-0 (PMC11890619; doi:10.1038/s42003-025-07835-0)
Supplement: Supplementary file 2 — Reporting Summary [file 42003_2025_7835_MOESM2_ESM.pdf]

nature portfolio

Corresponding author(s): Dr Razia Zakarya
D/Prof Brian Oliver
Prof Chris O'Neill

Last updated by author(s): Feb 11, 2025

Reporting Summary

Nature Portfolio wishes to improve the reproducibility of the work that we publish. This form provides structure for consistency and transparency in reporting. For further information on Nature Portfolio policies, see our Editorial Policies and the Editorial Policy Checklist.

Statistics

For all statistical analyses, confirm that the following items are present in the figure legend, table legend, main text, or Methods section.

n/a	Confirmed	
☐	☒	The exact sample size (*n*) for each experimental group/condition, given as a discrete number and unit of measurement
☐	☒	A statement on whether measurements were taken from distinct samples or whether the same sample was measured repeatedly
☐	☒	The statistical test(s) used AND whether they are one- or two-sided<br>*Only common tests should be described solely by name; describe more complex techniques in the Methods section.*
☐	☒	A description of all covariates tested
☐	☒	A description of any assumptions or corrections, such as tests of normality and adjustment for multiple comparisons
☐	☒	A full description of the statistical parameters including central tendency (e.g. means) or other basic estimates (e.g. regression coefficient) AND variation (e.g. standard deviation) or associated estimates of uncertainty (e.g. confidence intervals)
☐	☒	For null hypothesis testing, the test statistic (e.g. *F*, *t*, *r*) with confidence intervals, effect sizes, degrees of freedom and *P* value noted<br>*Give P values as exact values whenever suitable.*
☒	☐	For Bayesian analysis, information on the choice of priors and Markov chain Monte Carlo settings
☒	☐	For hierarchical and complex designs, identification of the appropriate level for tests and full reporting of outcomes
☐	☒	Estimates of effect sizes (e.g. Cohen's *d*, Pearson's *r*), indicating how they were calculated

Our web collection on statistics for biologists contains articles on many of the points above.

Software and code

Policy information about availability of computer code

Data collection	Details for all open source code used included in methods section
Data analysis	RNA-seq data: Trimmomatic (version 0.39), Bowtie2 (version 2.5.1), SAMtools (version 1.17), (DESeq) using SeqMonk (v1.48.1; Babraham Institute)<br>WGMS: DRAGEN Methylation Pipeline (v3.10), Seqmonk (v1.48.1; Babraham Institute)

For manuscripts utilizing custom algorithms or software that are central to the research but not yet described in published literature, software must be made available to editors and reviewers. We strongly encourage code deposition in a community repository (e.g. GitHub). See the Nature Portfolio guidelines for submitting code & software for further information.

Data

Policy information about availability of data

All manuscripts must include a data availability statement. This statement should provide the following information, where applicable:

- Accession codes, unique identifiers, or web links for publicly available datasets
- A description of any restrictions on data availability
- For clinical datasets or third party data, please ensure that the statement adheres to our policy

Data are publicly available in a repository.
Numerical source data for graphs and charts uploaded to Figshare and are available at: https://doi.org/10.6084/m9.figshare.28386518.v1111.

The RNA-Seq & WGMS raw and processed data files generated during and analysed during the current study are available in the NCBI Gene Expression Omnibus (GEO) repository with the following accession codes:
RNA-Seq:
GSE285128 (https://www.ncbi.nlm.nih.gov/geo/query/acc.cgi?acc=GSE285128)
WGMS:
GSE285243 (https://www.ncbi.nlm.nih.gov/geo/query/acc.cgi?acc=GSE285243)

Research involving human participants, their data, or biological material

Policy information about studies with human participants or human data. See also policy information about sex, gender (identity/presentation), and sexual orientation and race, ethnicity and racism.

Reporting on sex and gender	Study did not involve human participants, their data, or biological material
Reporting on race, ethnicity, or other socially relevant groupings	Study did not involve human participants, their data, or biological material
Population characteristics	Study did not involve human participants, their data, or biological material
Recruitment	Study did not involve human participants, their data, or biological material
Ethics oversight	Study did not involve human participants, their data, or biological material

Note that full information on the approval of the study protocol must also be provided in the manuscript.

Field-specific reporting

Please select the one below that is the best fit for your research. If you are not sure, read the appropriate sections before making your selection.

☒ Life sciences ☐ Behavioural & social sciences ☐ Ecological, evolutionary & environmental sciences

For a reference copy of the document with all sections, see nature.com/documents/nr-reporting-summary-flat.pdf

Life sciences study design

All studies must disclose on these points even when the disclosure is negative.

Sample size	Sample sizes were chosen to ensure WGMS data coverage sufficient enough to detect methylation differences as little as 5%.
Data exclusions	No data were excluded from the analysis except in the instance of low quality. Examples include replicates with poor biological quality or low NGS coverage. In such instances, a biologically and technically suitable replicate was used as replacement when available.
Replication	Biological and technical replicates where feasible.
Randomization	Allocations were not randomised. Covariates such as sex, original litter, litter size, litter sex skew, and data coverage were included in analyses when appropriate.
Blinding	All researchers were aware of the group allocation at all stages of the experiment, with the exception of histological analysis wherein slides were blinded until after images were quantified.

Reporting for specific materials, systems and methods

We require information from authors about some types of materials, experimental systems and methods used in many studies. Here, indicate whether each material, system or method listed is relevant to your study. If you are not sure if a list item applies to your research, read the appropriate section before selecting a response.

Materials & experimental systems

n/a	Involved in the study
☒	☐ Antibodies
☒	☐ Eukaryotic cell lines
☒	☐ Palaeontology and archaeology
☐	☒ Animals and other organisms
☒	☐ Clinical data
☒	☐ Dual use research of concern
☐	☐ Plants

Methods

n/a	Involved in the study
☒	☐ ChIP-seq
☒	☐ Flow cytometry
☒	☐ MRI-based neuroimaging

Animals and other research organisms

Policy information about studies involving animals; ARRIVE guidelines recommended for reporting animal research, and Sex and Gender in Research

Laboratory animals	BALB/c mouse. Male & Female, experiment commenced at 7 weeks of age (dams) and endpoint at 13-weeks (offspring)
Wild animals	Study did not involve wild animals
Reporting on sex	NGS findings only apply to female sex. Biological outcomes measured in both male and female sex, with stronger effect shown in females.
Field-collected samples	Study did not involve samples collected from the field
Ethics oversight	University of Sydney Animal Research Ethics Committee (ETH18-3175)

Note that full information on the approval of the study protocol must also be provided in the manuscript.

Plants

Seed stocks	No seed stocks or plant material used in this study.
Novel plant genotypes	No seed stocks or plant material used in this study.
Authentication	No seed stocks or plant material used in this study.
